# In Situ Measurement of the Electronic Structure of Oxidized Graphene Employing Quantum-Rate Spectroscopy

**DOI:** 10.1021/acsmeasuresciau.6c00179

**Published:** 2026-10-07

**Authors:** Edgar F. Pinzón, Leandro Hostert, Gabriela Dias da Silva, Paulo R. Bueno

**Affiliations:** † Department of Physics and Mathematics, Institute of Chemistry, 153997 São Paulo State University (UNESP), Araraquara, São Paulo 14800-060, Brazil; ‡ Centro Interdisciplinar de Química e Bioloxía (CICA) and Departamento de Química, Facultade de Ciencias, Universidade da Coruña, A Coruña, Galicia 15071, Spain

**Keywords:** oxidized graphene, quantum-rate spectroscopy, quantum capacitance, electronic structure, density of states, X-ray photoelectron spectroscopy, density functional theory

## Abstract

Measuring the electronic structure and defect states of oxidized single-layer graphene (ox-SLG) is essential for using graphene-based materials in commercial devices. In this work, we introduce quantum-rate spectroscopy (QRS), an electronic spectroscopic method based on the principles of low-energy quantum electrodynamics, to measure the electronic structure of ox-SLGs. QRS measures the quantum capacitance of ox-SLGs in an electrolyte environment, enabling the determination of the density of states (DOS) *in situ* and at room temperature. This methodology not only characterizes how oxygenated functional groups modify the pristine electronic structure of graphene by resolving defect-state energies with a precision comparable to the peak-position precision of X-ray photoelectron spectroscopy (XPS) performed under high-vacuum conditions but also enables direct correlation of the DOS measured by QRS with simulations from density functional theoretical methods. Notably, this agreement suggests that epoxy groups are the dominant defects, a finding that is corroborated by Raman spectroscopy and XPS analyses. Overall, QRS emerges as a suitable and mild-condition technique for the *in situ* characterization of ox-SLG structures.

## Graphical abstract

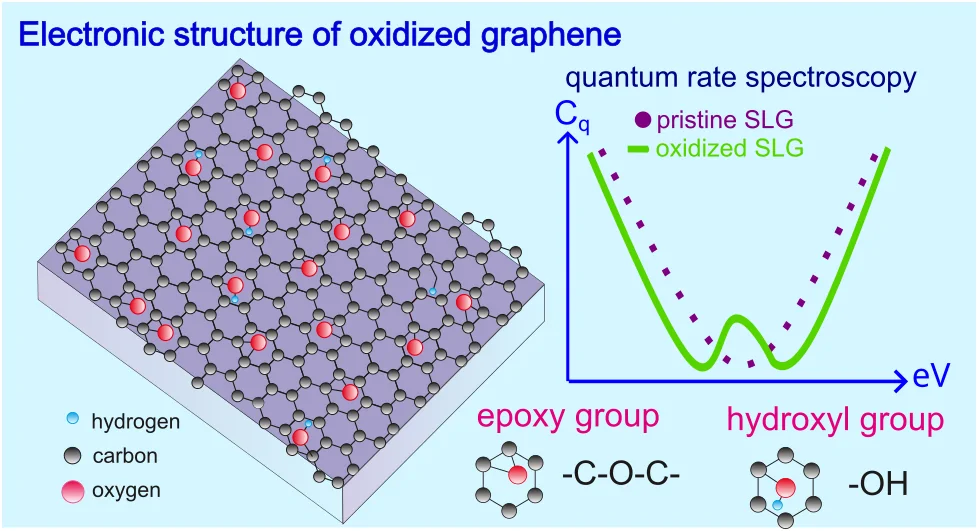

## Introduction

Graphene is referred to as a material structure formed of single sheets of carbon atoms, i.e., a monolayer of carbon atoms arranged in a honeycomb-like hexagonal lattice, derived from graphite.^1^ It has several potential applications. Nonetheless, accessibility to high-quality commercial samples has limited real-world device applications. For instance, a survey of commercially available graphene samples^1^ demonstrated that the majority of available graphene materials exhibit a high degree of undesirable oxidation. The oxidized graphene structure has been known as “graphene oxide”, a term that is controversial but intended to describe the controlled oxidation of graphene sheets.^2^ Oxidized single-layer graphene structures will here be referred to as ox-SLGs.

### Graphene and Oxidized Graphene Structures

Nanoscale electronics developed using graphene structures have attracted significant attention^3,4^ due to their potential applications in chemical and biological sensing,^5^ water desalination,^6,7^ supercapacitors,^8,9^ light emitters,^10,11^ and more. In response to these promising applications, both theoretical and experimental approaches have been widely used to predict and measure the electronic structure of this family of carbonaceous materials.^12–14^ Notably, the performance and operational capability of graphene-based devices primarily depend on the pristine electronic properties of the graphene employed as their fundamental building block.^12,15^

The V-shaped electronic structure of graphene has been extensively studied,^16,17^ including via computational approaches such as density functional theory (DFT)^18^ and tight-binding calculations.^19,20^ Likewise, experimental methods such as angle-resolved photoemission spectroscopy (ARPES)^21^ and near-edge X-ray absorption fine-structure (NEXAFS) spectroscopy^13^ have been employed to characterize the band structure, while electrical techniques have provided information about the electronic structure by measuring the quantum capacitance,^17,22,23^ which is directly related to the electronic density of states (DOS). For instance, some studies have demonstrated the ability to measure, with time-dependent electrochemical techniques, the V-shaped electronic structure obtained from quantum capacitance and conductance measurements,^23,24^ revealing a band structure resolution comparable to ARPES and DFT calculations.^24^

The electrodynamics arising from graphene’s V-shaped electronic structure highlights its significance in fundamental physics. At the minimum of this V-shape, known as the Dirac point, graphene’s electronic properties show relativistic characteristics, with charge carriers exhibiting ballistic transport and having zero rest mass.^13,16,25^ As a result, charge motion in this V-shaped structure can be described by quantum electrodynamics using Dirac, rather than Schrödinger, wave mechanics.^12^

The special quantum properties of graphene make it a useful solid-state model for studying relativistic quantum physics. These properties come from graphene’s highly symmetric carbon lattice, but real-world uses often need chemical changes. For example, chemical functionalization is necessary to attach receptor molecules for biosensing, while introducing atomic defects alters graphene’s electronic structure, which is crucial for gas sensing and optoelectronic devices.^26–28^

Oxidizing graphene is a key method for modifying its structure by adding oxygenated groups, such as epoxy, hydroxyl, and carbonyl, as shown in Figure 1. This is one of the most widely studied ways to covalently functionalize graphene.^14,29,30^ Oxidation can be achieved by exposing graphene to high temperatures (> 500 ^◦^C) in oxygen, reacting it with strong oxidizers,^31,32^ or applying an oxidation potential electrochemically.^33,34^ In all cases, oxidation forms new carbon–oxygen bonds, shifting carbon atoms from planar sp^2^ to distorted sp^3^ hybridization. These changes create electronic defects and gap states, strongly affecting graphene’s electronic properties.^29^

**Figure 1:**
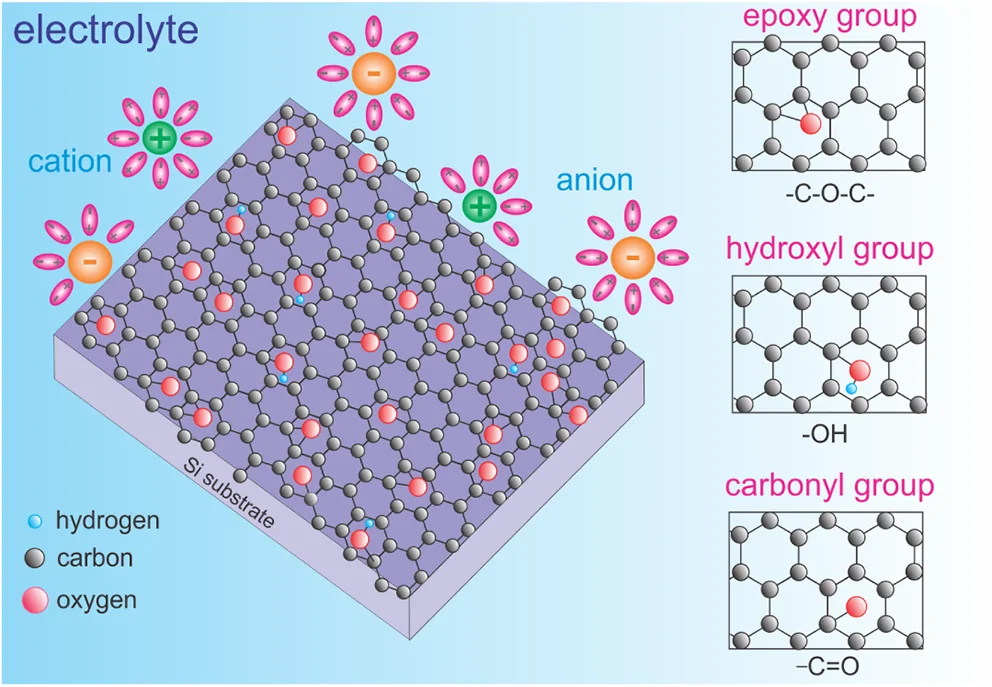
Schematic representation of an oxidized graphene surface embedded in an electrolytic medium. Oxidation primarily forms epoxy, hydroxyl, and carbonyl functional groups on the pristine graphene structure. By probing the electronic states associated with these oxygen-containing moieties via electrochemical measurements (e.g., EIS), ions in the electrolytic medium shield the electron’s charge at the surface.

The extent and nature of these oxidation-induced changes depend on specific process conditions. Important factors include temperature, type of oxidizing agent, magnitude of oxidation potential, and the pH of the electrolytic environment. As a result, oxidized graphene surfaces exhibit different oxidation levels, varying according to the type, concentration, and spatial arrangement of oxygenated groups. These differences lead to distinct electronic structures. This creates a challenge in producing reliable ox-SLGs, as it involves controlling and quantifying sp^3^ defects.^14,29,30^ A deeper understanding of how these defects affect electronic structure is essential to optimize graphene’s electronic properties for specific applications.

A combination of experimental techniques and computational chemistry calculations has addressed the challenges of characterizing the oxidized graphene surfaces. For instance, experimental methods such as X-ray photoelectron spectroscopy (XPS), high-resolution solid-state carbon nuclear magnetic resonance (C-NMR),^29,35^ and electron energy loss spectroscopy (EELS) have collectively demonstrated that oxidized graphene surfaces primarily consist of residual sp^2^ carbon domains interspersed with epoxy and hydroxyl groups.^14^ In parallel, complementary first-principles calculations using DFT have provided insights into energetically favorable arrangements of these oxygenated groups. Specifically, these studies indicate that hydroxyl and epoxy groups tend to cluster into specific stripe-like formations, interspersed with sp^2^ carbon regions, in a manner that minimizes energy. Furthermore, by considering different concentrations and arrangements of oxygenated groups, computational models have revealed the oxidation effects on the DOS of graphene.^30,36^

Despite the advances, existing computational models remain idealized, as they typically consider a reduced number of carbon atoms in the graphene sheet and do not fully account for inherent atomic defects, such as vacancies, edge effects, or interactions arising from high densities of oxygenated groups. These limitations hinder the ability to predict the properties and performance of oxidized graphene, underscoring the need for experimental approaches capable of performing an *in situ* analysis of the electronic structure of oxidized graphene-based materials. While ARPES and NEXAFS provide highly accurate resolution of the electronic structure, these techniques cannot perform *in situ* characterization because of their reliance on low temperatures and ultrahigh vacuum. Additionally, they require expensive equipment and extensive operator training, making them impractical for routine characterization.

To overcome these challenges, this work introduces an experimental approach for the *in situ* measurement of the electronic DOS of oxidized graphene materials that employs time-dependent electrical perturbation in an electrolyte at room temperature and that uses a low-cost, hand-held electronic apparatus that has been referred to as quantum-rate spectroscopy (QRS).^24,37^ QRS is a hand-held spectroscopic methodology that enables rapid and practical characterization of graphene’s electronic properties.^24^ In the present QRS applicability study, we compare the results with those of other traditional spectroscopic techniques and computational simulations, providing a proof-of-concept for using QRS methods in the analysis of chemical and electronic properties of oxidized single-layer graphene-based compositions.

Before presenting a detailed QRS characterization of ox-SLGs, a brief overview of the principles and concepts of quantum electrochemistry that underpin this methodology is given in the following section.

### Quantum-Rate Theory

The QRS method is grounded in quantum electrodynamics principles and permits the experimental analysis of the electronic structure through the time-dependent electric perturbation of the scrutinized material confined to the surface of an electrode embedded in an electrolyte medium. To contextualize the principles of the method, we must first introduce the theoretical framework from which it is derived, namely, quantum rate theory.

Describing electrodynamics within molecular and nanoscale structures—particularly due to their significant chemical and biological importance—requires an understanding of electron transfer (ET) and coherent electron transport phenomena. Such understanding significantly impacts comprehension of redox reaction rates and nanoscale electronics.^38^ For example, quantum electrodynamics phenomena not only explain ET reaction rates^38^ but also quantify coherent electronic communication between electrode states and donor and acceptor moieties in push-pull *π*-conjugated heterocyclic structures.^39^ Despite differences in underlying mechanisms, a broad range of electrodynamic processes in an electrolyte environment are described by a characteristic quantum mechanical time or rate constant. This forms the basis for explaining low-energy quantum electrodynamics phenomena in electrolytes, referred to as quantum-rate (QR) theory.^38,40,41^

QR theory defines a characteristic quantum rate, denoted by *ν*, as the ratio between the quantum conductance *G* and electrochemical capacitance *C*_*μ*_, such that

1 $$\begin{array}{rr} & \nu =\frac{G}{C_{\mu }}=G_{0}\overset{N}{\underset{n=1}{\sum }}T_{n}\left(\mu \right)\left(\frac{1}{C_{e}}+\frac{1}{C_{q}}\right)=\\ & \frac{g_{s}}{h}\overset{N}{\underset{n=1}{\sum }}T_{n}\left(\mu \right)\left(\frac{e^{2}}{C_{e}}+\frac{e^{2}}{C_{q}}\right)=\\ & \frac{g_{s}}{h}\overset{N}{\underset{n=1}{\sum }}T_{n}\left(\mu \right)\left(E_{\mu }\right) \end{array}$$

in which $G=G_{0}\overset{N}{\underset{n=1}{\sum }}T_{n}\left(\mu \right)$ . Here, $\overset{N}{\underset{n=1}{\sum }}T_{n}\left(\mu \right)$ represents the sum of transmission probabilities *T*_*n*_(*μ*) for each quantum channel *n* at chemical potential *μ*, characterizing the likelihood of electron transmission through those channels under a chemical potential difference of d*μ* = −*e*d*V*,^38,42^ where d*V* is the electric potential difference between the two quantum energy levels involved. The constant *G*_0_ = *g*_s_ *e* ^2^/*h* ≈ 77.5 μS is the conductance quantum, where *g*_s_ is the spin degeneracy, *e* is the elementary charge, and *h* is the Planck constant. In an ideal case where all *T*_*n*_(*μ*) = 1, the sum $\overset{N}{\underset{n=1}{\sum }}T_{n}\left(\mu \right)$ equals *N*, and thus the conductance *G* attains *NG*_0_, indicating perfect and coherent electron transport without scattering.^40,43^ The term *E*_*μ*_ = *e* ^2^/*C*_e_ + *e* ^2^/*C*_q_ in the last part of eq 1 denotes the electrochemical energy, with *C*_e_ and *C*_q_ representing electrostatic and quantum capacitances, respectively; this term will be discussed further later.

The electrochemical capacitance *C*_*μ*_ term of eq 1 is modeled as a series combination of two distinct capacitance contributions. These are the electrostatic *C*_e_ and quantum *C*_q_ capacitances, leading to 1/*C*_*μ*_ = 1/*C*_e_ + 1/*C*_q_. Although both contributions relate to charge storage phenomena, their physical origins differ markedly. For instance, *C*_e_ arises from the Coulombic phenomenon and is governed by the system’s geometric configuration. It can be modeled using classical capacitance settings, such as parallel-plate and electrical double-layer models. Conversely, *C*_q_ reflects the occupancy dynamics of quantum electronic states, such as charge dynamics between donor and acceptor state levels. Consequently, *C*_q_ is intimately related to the system’s electronic structure and is defined by *C*_q_ = *e* ^2^(d*n*/d*E*), where d*n*/d*E* is the electronic DOS.^38,41,44^

When a molecule or nanoscale object is placed in a salt solution and exposed to a changing electric field, its quantum states can gain or lose charge. This lets us examine its electronic states. The surrounding ions in the solution shield or screen these quantum states, so the system remains electrically neutral^45,46^ (see Figure 1). Because of this screening, the electrostatic and quantum energy contributions become nearly indistinguishable since *C*_*e*_ ≈ *C*_*q*_. The total electrochemical energy then simplifies to *E*_*μ*_ = *e* ^2^/*C*_*μ*_ and *e* ^2^/*C*_*μ*_ = 2*e* ^2^/*C*_*q*_ = 2*e* ^2^/*C*_*e*_. The factor of 2 shows an energy degeneracy *g*_e_ between the two energy contributions. Following these QR theory ideas, *ν* in eq 1 becomes *ν* = *g*_s_ *g*_e_(*e* ^2^/*hC*_q_) = *g*_s_ *g*_e_(*E*_q_/*h*).

Observe that this outcome for *ν* follows a linear relationship between *E* and *ν*, i.e., *E* ∝ *hν*, and hence aligns with the Planck–Einstein relation, an unconditional expression in the formulation of quantum electrodynamics.^39,41,47^ Note that, with QR theory premises, *ν* can be simplified and stated only in terms of quantum RC circuit element, i.e., *ν* = *g*_e_/*R*_q_ *C*_q_ = 1/*τ*, where *R*_q_ = 1/*G* is the quantum resistance and *τ* = *R*_q_ *C*_q_ is a characteristic time constant stated as the reciprocal of *ν*. This permits, as will be shown later, the analysis of quantum electrodynamics phenomena within an electrolyte environment using a quantum RC circuit analysis.

Hence, QR theory, derived from the interplay between *G* and *C*_*μ*_, is a quantum coherent phenomenon that has proved effective in modeling various phenomena. For instance, it accounts for the rate of ET reactions in diffusionless settings.^38,44^ In addition, QR theory has been applied to the quantum electrodynamics of graphene.^24,40^ It also describes the electronic communication of donor and acceptor states within organic heterocyclic systems^39^ in connection with electrode states. Furthermore, QR theory addresses the long-range ET involved in the respiration process of Geobacter sulfurreducens films,^41^ and it explains the quantum mechanical origin of the supercapacitance phenomenon observed in reduced graphene oxide.^47^

Building upon these theoretical principles, QR theory has promoted the development of experimental methodologies for the *in situ* electroanalysis of the electronic structure of nanoscale structures in electrolytic environments, enabling the measurement of the electronic DOS of inorganic^48^ and organic semiconducting films,^39^ redox-active monolayers,^43,44,49^ inorganic quantum dots,^37^ and single-layer graphene.^24^ The application of QR theory principles is important for developing quantum electroanalytical methods.^50^ For instance, they have been employed in the development of highly sensitive (between femtomolar and attomolar ranges) immuno-^51^ and genosensors.^52^

The application of QR theory principles for developing the aforementioned *in situ* spectroscopic capability for characterizing the electronic structure of various moieties is discussed in the next section.

### Quantum-Rate Spectroscopy

The electronic spectroscopic nature of QRS is based on *E*_*e*_ ≈ *E*_*q*_, implying that the dynamics of the solvent/electrolyte rule the quantum electrodynamics owing to classical and quantum energies being degenerate or overlapped, leading to a low-energy quantum electrodynamics phenomenon such that (d*E*_*μ*_/d*n*) = *g*_e_(*e* ^2^/*C*_q_) = *g*_e_(*e* ^2^/*C*_e_), which is equivalent to *C*_q_ = *g*_e_ *e* ^2^(d*n*/d*E*_*μ*_), where (d*n*/d*E*_*μ*_) is identified as the electrochemical DOS.

The electronic communication between an electrode (as an experimental probe) and the electrochemical DOS follows quantum electrodynamics principles (particle-wave duality) governed by the Dirac equation, such that^40^

2 $$\left(\frac{dE_{\mu }}{dn}\right)=g_{e}\left(\frac{e^{2}}{C_{q}}\right)=\hbar c_{*}\cdot k$$

where c_*_ denotes the Fermi velocity and **k** represents the electron’s wavenumber. From eq 2, it follows that d*E*_*μ*_ = ℏc_*_·**k** d*n*, an energy-state relationship that can be perturbed in a time-dependent way, such as

3 $$\left(\frac{dE_{\mu }}{dt}\right)=\hbar c_{*}\cdot k\left(\frac{dn}{dt}\right)$$

where both *E*_*μ*_ and *n* are time-dependent variables, i.e., (d*E*_*μ*_/d*t*) = *E*_*μ*_(*t*) and (d*n*/d*t*) = *n*(*t*). This formulation describes a spectroscopic principle whereby the application of a time-varying energy input, *E*_*μ*_(*t*), to an electrochemical system elicits a corresponding response, *n*(*t*), related to the quantum states.

To further analyze this electrodynamics phenomenon, let us consider harmonic oscillatory perturbations and their respective responses. In this case, the excitation and response functions can be expressed as *E*_*μ*_(*t*) = *E̅*_*μ*_ + *Ẽ*_*μ*_ and *n*(*t*) = *n̅* + *ñ*, where *Ẽ*_*μ*_ = *E*_*μ*,0_ exp­(*jωt*) and *ñ* = *n*_0_ exp­(*jωt* – *ϕ*), with *ω* being the angular frequency and *ϕ* the phase difference between the two signals. *E*_*μ*,0_ and *n*_0_ are the energy perturbation and state occupancy response amplitudes, respectively. To connect these definitions and advance our analysis, we now formulate a complex energy-state function as

4 $$E_{\mu }^{*}\left(\omega \right)=\left[\frac{dE_{\mu }\left(t\right)}{dn\left(t\right)}\right]=\left(\frac{E_{\mu ,0}}{n_{0}}\right)\exp\left(j\phi \right)$$

where the real component of this function is given by

5 $$R\left[E_{\mu }^{*}\left(\omega \right)\right]=\left(\frac{E_{\mu ,0}}{n_{0}}\right)R\left[\exp\left(j\phi \right)\right]=\hbar c_{*}\cdot k$$

By considering a frequency *ω* → 0, which characterizes equilibrium between the electronic structure and the surrounding environment’s harmonic oscillatory perturbation, we can define a limiting condition: *E*_*μ*,0_ = *g*_e_ *n*_0_(*e* ^2^/*C*_q_) = ***ℏ*** c_*_ · **k**.^37,38,41^ This allows access to the intrinsic electronic structure via time-dependent electric and equivalent circuit analysis. We will further demonstrate this in the Quantum RC Circuit Analysis section, where the equilibrium condition *ω* → 0 makes it possible to measure a *C*_*q*_ value that defines *E*_*μ*,0_.

The theoretical principles underlying QRS will be applied to measure the electronic structure of ox-SLG structures, and comparisons will be made with traditional XPS and computational chemistry analyses.

## Materials and Methods

### Reagents and Solutions

Pristine single-layer graphene (SLG), synthesized via chemical vapor deposition and supplied by Graphenea on a 90 nm SiO_2_ layer over a silicon wafer substrate, was used for various spectroscopic analyses in this work. Metallic contacts were fabricated on these SLGs using radio frequency (RF) sputtering with a wetting layer approach: first, a 10 nm titanium layer was deposited to enhance adhesion, followed by a 100 nm gold layer.

Ionic liquid [BMIM]­BF_4_ (1-butyl-3-methylimidazolium tetrafluoroborate) from Sigma-Aldrich was employed as the electrolyte and obtained with a purity of >98%. A phosphate buffer (PB, pH 3) with a concentration of 12 mmol L^–1^ using 0.2 M KNO_3_, 10 mM Na_2_HPO_4_ · 12H_2_O, and 2 mM KH_2_PO_4_ (Sigma-Aldrich) was prepared. The pH of the solution was adjusted using 0.1 M HNO_3_. Milli-Q water (Millipore system, 18.2 MΩ·cm at 25 °C) was used to prepare the solutions and electrolytes in the present work.

### Oxidation of Single-Layer Graphene

The oxidation of SLGs was performed using an electrochemical approach in a three-electrode cell setup. Specifically, the graphene surface served as the working electrode, a platinum wire was used as the counter electrode, and Ag|AgCl (in 3 M KCl) functioned as the reference electrode. Following cell assembly, the electrolyte used for electrochemical modification was a phosphate buffer (PB) solution at pH 3. With the setup prepared, the oxidation of SLGs was conducted using the chronoamperometry technique by applying constant potential pulses of 1.7 V for 45 s each. Specifically, six pulses were applied per device, separated by rest periods of 120 s at open circuit. The three independently prepared devices exhibited anodic charges of 169.25, 198.94, and 249.08 μC cm^–2^ during the final oxidation pulse, corresponding to an average of 205.8 ± 40.4 *μ*C cm^–2^ (RSD = 19.6%); no target charge or acceptance window was defined a priori, and this dispersion is reported as the batch-to-batch reproducibility of the protocol. A representative chronoamperogram and the charge passed for each device are provided in Section S10 of the Supporting Information. This sequence of steps resulted in oxidized SLGs, enabling the controllable chemical modification of the pristine SLG structure through the quantification of current and charge.

### Electrochemical Measurements

Electrochemical impedance spectroscopy (EIS) measurements were performed on both pristine and oxidized single-layer graphene (SLG) using a portable PalmSens4 potentiostat equipped with a frequency response analyzer (FRA) module. A two-terminal electrochemical cell was employed, where the working electrode was either a pristine or oxidized graphene monolayer. A platinum (Pt) foil served as both the counter electrode (CE) and quasi-reference electrode (RE) by short-circuiting the CE and RE terminals. All experiments were carried out in [BMIM]­BF_4_. EIS data were collected over a frequency range from 1 MHz to 0.05 Hz, using a root mean square (RMS) voltage amplitude of 10 mV and a fixed bias at the open-circuit potential (OCP). Additionally, EIS measurements at the fixed low-frequency limit (where the Nyquist plot semicircle closes at OCP) were conducted in sweeping mode, with the potential varied from −1.5 V to 0.5 V versus Pt. The stability of the Pt quasi-reference electrode was assessed through the reproducibility of the open-circuit potential across the three independently prepared interfaces, which agreed within ±21 mV for pristine SLG and ±65 mV for ox-SLG; these values, which conservatively bound both electrode-to-electrode variability and drift over the measurement series, were adopted as the potential-axis uncertainty. This uncertainty, together with the 10 mV RMS perturbation amplitude, was propagated into all potential-axis (energy) quantities reported in this work (see Section S8 of the Supporting Information). All uncertainties reported in this work correspond to three independent replicates, each defined as a separately fabricated graphene device (separate chip, separate contact deposition, separate oxidation run) measured in a fresh electrolyte aliquot.

From the raw EIS data, a Nyquist capacitive plot can be derived using the real (*C*′) and imaginary (*C*″) components of complex capacitance. These components are obtained via the relation C*­(*ω*) = 1/jωZ*­(*ω*), where Z*­(*ω*) and C*­(*ω*) represent the complex impedance and capacitance functions, respectively. Here, *ω* is the angular frequency, and $j=\sqrt{-1}$ is the imaginary unit. Accordingly, the complex capacitance can be expressed as C*­(*ω*) = *C*′ + *jC*″. The real component *C*′ is given by *Z*″/(*ω*|*Z*|^2^), and the imaginary component *C*″ by *Z*′/(*ω*|*Z*|^2^). In these equations, *Z*′ and *Z*″ denote the real and imaginary components of the impedance function Z*­(*ω*) = *Z*′ – *jZ*″, while |*Z*| is the impedance modulus. The function C*­(*ω*) is particularly relevant in QR theory, as it enables the understanding of electronic relaxation processes that stem from interactions between quantum states. It is expressed as

6 $$C^{*}\left(\omega \right)=\frac{C_{q}}{1+\left(j\omega \tau \right)}∼C_{q}\left(1-j\omega \tau \right)+O\left(\omega^{2}\right)$$

where *C*_*q*_ represents the low-frequency equilibrium capacitive response and, under the premises of QR theory, corresponds to the quantum capacitance of the nanoscale structure attached to the electrode. The electronic relaxation described by eq 6 is characterized by the relaxation time *τ*, which is related to the quantum rate *ν* as *τ* = 1/*ν* = *R*_q_ *C*_q_, where *R*_q_ denotes the quantum resistance.

### Spectroscopic Characterization

XPS analysis was performed at the multiuser facility of the Institute of Chemistry, São Paulo State University (UNESP, Araraquara), using a commercial UNI-SPECS ultra-high-vacuum (UHV) spectrometer system, operating at pressures below 5 × 10^–7^ Pa. Non-monochromatized electromagnetic radiation from Al K*α* (1486.6 eV) and Mg K*α* (1253.6 eV) was used as the source of electronic excitation, and the analyzer pass energy was set to 10 eV. To enhance accuracy, the Shirley method was used to eliminate the inelastic contribution within the high-resolution spectra. The atomic percentage composition of the subsurface layer (≤5 nm) was ascertained by evaluating the relative area proportions of the corrected spectra, factoring in the atomic sensitivity coefficients established by Scofield. This determination possessed an accuracy of ± 5%. The spectral profiles underwent deconvolution using Voigtian-type functions, which involved a combination of Gaussian (70%) and Lorentzian (30%) components. The full width at half maximum (FWHM) of the peaks ranged from 1.2 to 2.1 eV, while the peak positions were pinpointed with a precision of ± 0.1 eV.

Raman spectroscopic measurements were performed using a Micro Raman spectrometer (LabRAM HR, Horiba Jobin Yvon) coupled with an optical microscope, featuring a spatial resolution of 1 μm^2^ (objective lens of 50× magnification). The spectral range analyzed was from 1200 to 3000 cm^–1^. A 29 mW He–Ne laser (632.8 nm, 1.96 eV) was used. The recorded Raman spectra were baseline-corrected and deconvoluted using Lorentzian profiles.

### Computational Chemistry Simulations

Computational simulations modeled three systems to match experimental conditions. The basic SLG structure had 180 carbon atoms and was modified by adding 2, 9, or 18 oxygen atoms for low, medium, and high oxidation. The oxidation level denotes the oxygen-to-carbon atomic ratio, i.e., 1.1%, 5%, and 10%, labeled for brevity as the 1%, 5%, and 10% levels. One representative configuration of the oxygen-containing groups was employed at each oxidation level, with the groups distributed as uniformly as possible over the sheet (maximizing the distance between neighboring defects) to minimize artificial defect–defect interactions introduced by the finite supercell; accordingly, the calculated DOS profiles are used here as qualitative references for the position and emergence of defect-induced states, rather than as configuration-averaged predictions (see Section S3 of the Supporting Information for a discussion of this limitation and of the robustness of the features used for comparison with experiment). Geometry optimization was done until the minimum energy configuration was found. This energy and atomic layout reflect the system’s energy, which was calculated using spin-polarized density functional theory (DFT).^53,54^ All simulated systems converged to a non-magnetic state, with negligible spin splitting near the Fermi level. The SIESTA program^55^ was used for these calculations. The Generalized Gradient Approximation (GGA), with the Perdew–Burke–Ernzerhof (PBE) functional,^56^ handled the exchange–correlation effect. This method is based on earlier local formulations.^57,58^

After modeling setup and energy optimization, a periodic cubic simulation cell with dimensions of 22.11 Å, 21.27 Å, and 20.00 Å was used. Subsequently, the Kohn–Sham wave functions for valence electrons were expanded with a double-zeta polarized (DZP) basis set consisting of finite-range, numerically confined orbitals.^59^ Finally, norm-conserving pseudopotentials described the core–valence interaction.^60^

Building on these simulation settings, a real-space integration grid with an energy cutoff of 200 Ry was applied. Periodic boundary conditions were then set to simulate the extended system. Brillouin zone sampling was done using a Monkhorst–Pack grid. Subsequently, functionalization studies involving hydroxyl groups were conducted (see Section S2 of the Supporting Information).

For all calculations, the Fermi level for both pristine and oxidized SLGs was set at 0.0 eV. This modeling approach enabled comparisons between theoretical predictions for the electronic properties of SLGs at different oxidation levels and defect types and the experimental results. A data set collection of computational results is available in the ioChem-BD repository^85,86^ and can be accessed via https://iochem-bd.bsc.es/browse/handle/100/504758.

## Results and Discussion

This section investigates ox-SLG structures using conventional spectroscopic and computational simulation methods and compares them to the QRS approach. This analysis reveals how oxidation-induced chemical defects affect graphene’s electronic structure. Despite ongoing debates, few comparative experimental studies on graphene oxidation exist; therefore, these findings provide valuable electronic characterization of 2D carbonaceous structures. The section opens with Raman spectroscopy and XPS characterization.

### Spectroscopic Characterization

Raman spectroscopy was used to analyze the structure of both pristine SLG and ox-SLG samples, as shown in Figure 2. The Raman spectrum of pristine SLG displays two key features of monolayer graphene: the G band (1582 cm^–1^), from in-plane vibration of sp^2^ carbon atoms, and the 2D band (2633 cm^–1^). The high 2D-to-G intensity ratio (*I*_2D_/*I*_G_ ∼4.09) confirms the sample’s monolayer nature. Defect-related features include the D band (1350 cm^–1^) and a less intense D + D″ band (∼2450 cm^–1^). These bands show a low density of defects, likely from CVD growth or substrate transfer.^61–63^

**Figure 2:**
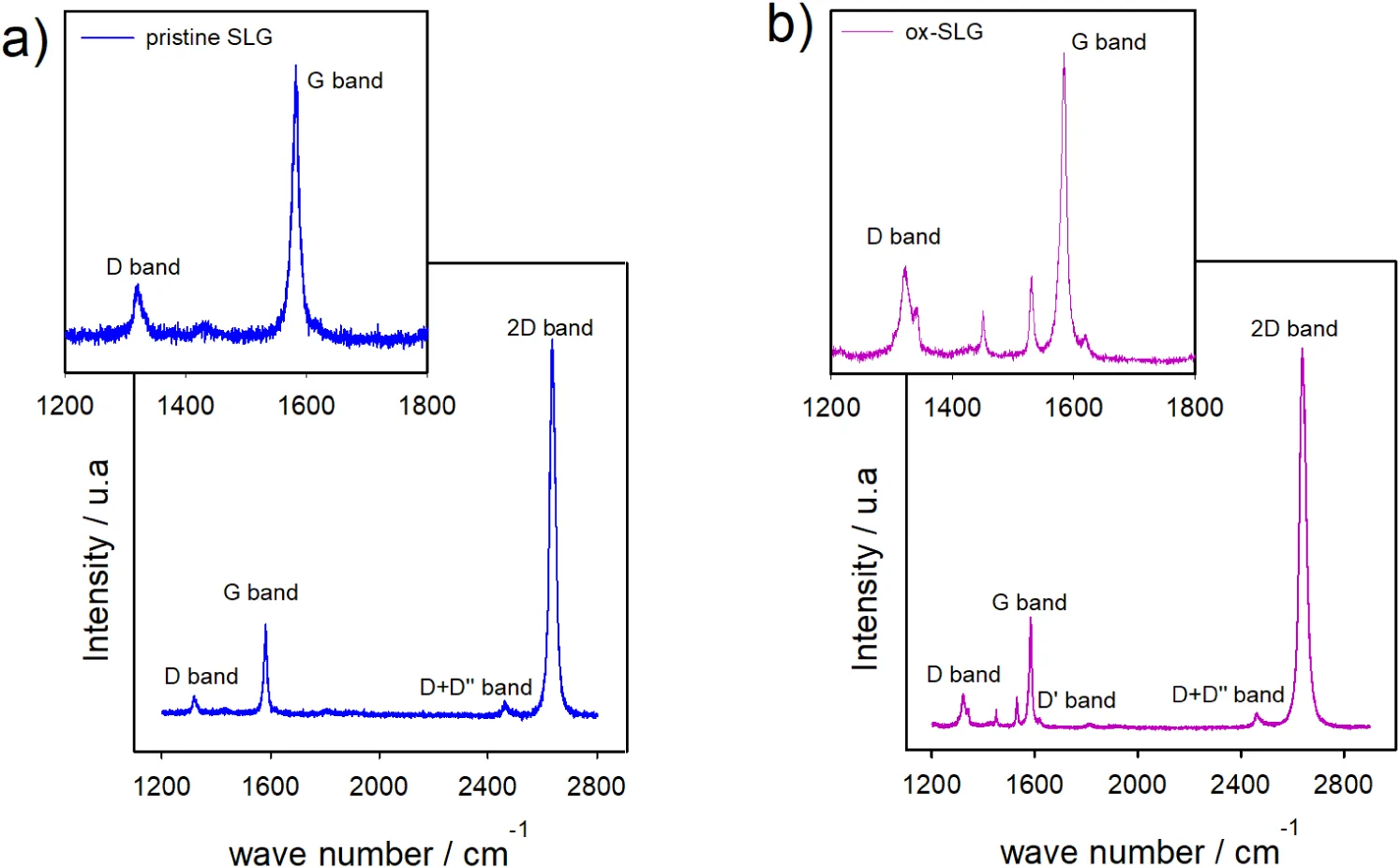
Raman spectra of (a) pristine SLG and (b) electrochemically oxidized SLG (ox-SLG). The oxidized sample shows an increased D band intensity and the emergence of new Raman features, indicating a higher defect density.

On the other hand, the Raman spectrum of ox-SLG in Figure 2b reveals clear signatures of electrochemical oxidation compared to pristine SLG. The most prominent change is the significant increase in the D band intensity (1350 cm^–1^),^64^ with the *I*_D_/*I*_G_ ratio rising from 0.17 in pristine SLG to 0.30 in ox-SLG for the representative spectra shown (the median over 12 locations is 0.28; see below), indicating an increased defect density.^65,66^ Additionally, the emergence of the D′ band and distinct Raman features between the D and G bands are attributed to disorder-induced vibrational modes. Together, these spectral features confirm successful oxidation, disruption of the sp^2^ network, and formation of oxygen-functionalized domains such as epoxy groups. Raman spectra were acquired at multiple ox-SLG surface points to assess local structural variation. Analysis of parameters such as *I*_D_/*I*_G_ at each measurement location is detailed in Section S4 of the Supporting Information. The box plot of *I*_D_/*I*_G_ values (see Figure S4) was generated from 12 localizations on ox-SLG. It shows a median of about 0.28, with an interquartile range of 0.26–0.30 (IQR ≈0.03–0.04). This indicates limited variability across the sampled regions. The whiskers range from about 0.24 to 0.43, and there are two outliers, at about 0.23 and 0.46. Overall, these results indicate macroscopically consistent defect density. However, the spread and the two outliers suggest localized surface variations.

The defect density was further quantified using the model proposed by Cançado et al.,^67^ which correlates the *I*_D_/*I*_G_ ratio to the inter-defect distance *L*_D_. A detailed description of this calculation appears in Section S5 of the Supporting Information. For an excitation wavelength of 632.8 nm (He–Ne laser) and an average *I*_D_/*I*_G_ ≈0.28, the estimated inter-defect distance is *L*_D_ ∼ 32 nm, corresponding to a defect density of about 10^10^ cm^–2^. This indicates that the ox-SLG surface is within the low-defect-density regime, where the graphene lattice remains largely preserved.

Moreover, a detailed characterization was performed using high-resolution X-ray photoelectron spectroscopy (XPS), focusing on the C 1s region, to further validate the oxidation process and identify oxygenated functional groups incorporated onto the graphene surface. Figure 3 *a* shows comparisons of the C 1s spectra of pristine and oxidized graphene structures. In both samples, a dominant peak at approximately 284.5 eV is observed, attributed to C–C bonding within the carbon network (sp^2^ C), as previously reported by Ruoff et al.^68^ This prominent feature confirms that the basal graphene structure is predominant and that its chemical integrity is maintained. Importantly, the relatively high contribution of epoxy moieties (C–O–C) observed even in the pristine SLG (Figure 3 *b*) should not be interpreted as an intrinsic chemical feature of the graphene lattice. Instead, this signal is predominantly associated with extrinsic effects arising from the substrate and the graphene–substrate interface, including surface functional groups of the dielectric support,^69^ adsorbed ambient contaminants,^70^ and interfacial charge-transfer phenomena.^29^ These contributions act as a background component in the XPS spectra and remain essentially independent of the oxidation treatment. This assignment is consistent with literature C 1s spectra of as-transferred CVD graphene on SiO_2_, which exhibit comparable C–O region intensity arising from transfer-polymer residues, adventitious adsorbates, and the silanol-terminated substrate surface sampled within the photoelectron information depth.^69,70^ Importantly, because this extrinsic contribution is constant between the two samples, it cancels in the pristine-versus-oxidized comparison, and all oxidation-related conclusions drawn here rest on the change of the spectra rather than on their absolute deconvolution. In contrast, the spectral changes induced by electrochemical oxidation, namely the slight shift of the main C 1s peak toward higher binding energies and its noticeable broadening (Figure 3 *c*), represent a genuine, intrinsic response of the graphene lattice to the incorporation of oxygenated functional groups. To provide detailed insight, deconvolution of the XPS spectra for both conditions (Figure 3 *b* and *c*) reveals additional peaks at 285.3, 286.4, and 288.9 eV, corresponding to −C–O–C– (epoxy), C=O (carbonyl), and O–C=O (carboxylic) functional groups, respectively. Following electrochemical oxidation, the systematic increase in the −C–O–C– component, clearly correlated with the pronounced rise in the *I*_D_/*I*_G_ ratio observed by Raman spectroscopy, unambiguously reflects the intrinsic functionalization of the graphene surface. Taken together, these findings demonstrate that, while substrate-related contributions define a constant extrinsic background, the observed spectral evolution is governed by oxidation-induced chemical modification of the graphene itself. A fully quantitative analysis—including the binding energies, FWHM, relative areas, and associated uncertainties of all fitted C 1s components for both samples—is provided in Section S9 of the Supporting Information. The epoxy (C–O–C) fraction of the C 1s envelope increases from 26.6 ± 0.2% to 48.9 ± 0.3% upon oxidation, at the expense of the sp^2^ C–C fraction (57.7 ± 0.1% → 47.0 ± 0.3%), quantitatively confirming epoxy groups as the dominant oxidation-induced functionality.

**Figure 3:**
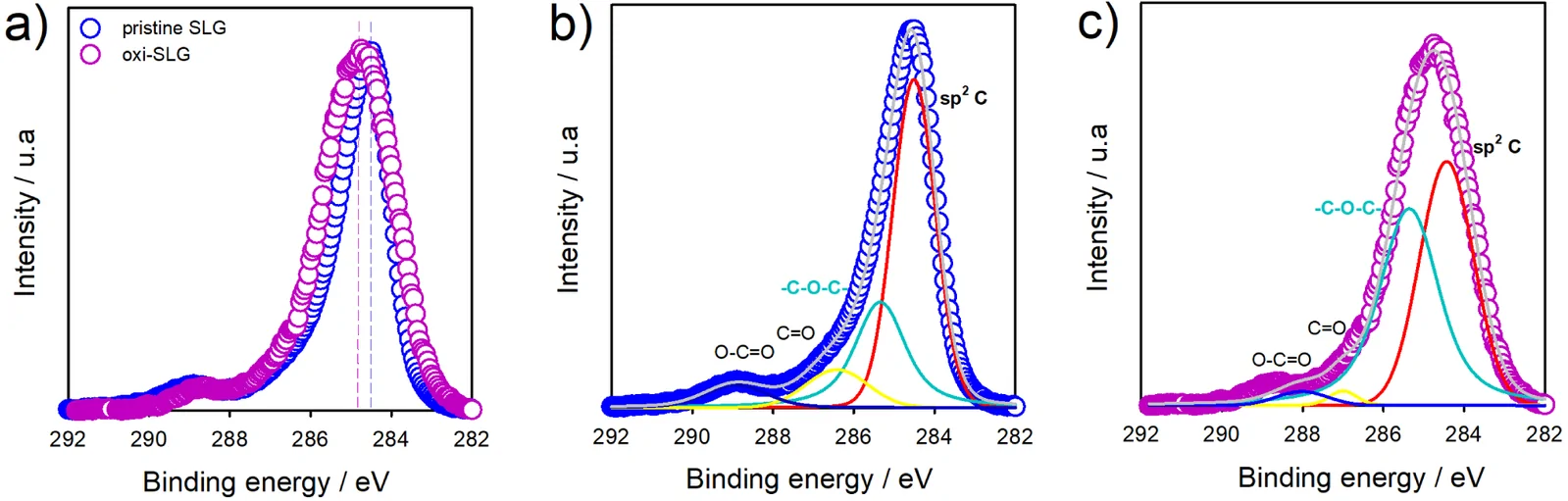
(a) XPS spectra recorded for both pristine and oxidized graphene. High-resolution C 1s core-level spectra with fitted component peaks for (b) pristine and (c) oxidized graphene.

Nevertheless, although Raman and XPS characterizations accurately describe structural features, they do not reveal how the incorporation of oxygenated groups influences the electronic structure or enable a quantitative estimation of their concentrations. The following sections discuss the experimental results obtained via QRS and interpret them within the framework of QR theory. These results demonstrate that we can measure the electronic DOS of the ox-SLG surface in situ, thereby enabling an investigation of how defect states affect its electronic properties. Moreover, analyzing EIS measurements from the perspective of quantum RC circuits provides insights into the electron transport regime that governs the electrodynamics of this graphene-derived structure.

### Quantum RC Circuit Analysis

Previous Raman and XPS analyses provided important insights into the structural and chemical changes in the SLG structure resulting from electrochemical oxidation. Building on these findings, an in-depth analysis was conducted on both pristine and oxidized SLG (ox-SLG) surfaces using QRS, which is based on time-dependent electrochemical measurements, along with quantum electrodynamic interpretations derived from the QR theory framework. To achieve this, impedance-derived electrochemical capacitance spectroscopy (ECS) measurements were conducted on both SLG interfaces using [BMIM]­BF_4_ as the electrolyte medium. It is important to note that these measurements were performed at the open-circuit potential (OCP, −0.254 ± 0.021 V vs Pt) of each interface. In this way, the analyses were conducted under zero-charge conditions, where no conductive current flows through the SLG structures. As demonstrated in previous studies, under these charging conditions, the electronic structure responses obtained from these measurements arise solely from displacement currents generated by the alternating current (AC) nature of the ECS technique.

Figure 4 *a* shows the complex capacitance C*­(*ω*) as a capacitive Nyquist plot, with *C*″ on the ordinate and *C*′ on the abscissa. The plot exhibits a single semicircle as per eq 6, indicating an intrinsic graphene relaxation process. In the low-frequency limit (*ω* → 0), C*­(*ω*) ∼ *C*_q_, allowing direct extraction of quantum capacitance *C*_q_ from the *C*′ value where the semicircle closes in Figure 4 *a*. The measured *C*_q_ is 1.435 ± 0.156 μF cm^–2^ (mean of three measurements).

**Figure 4:**
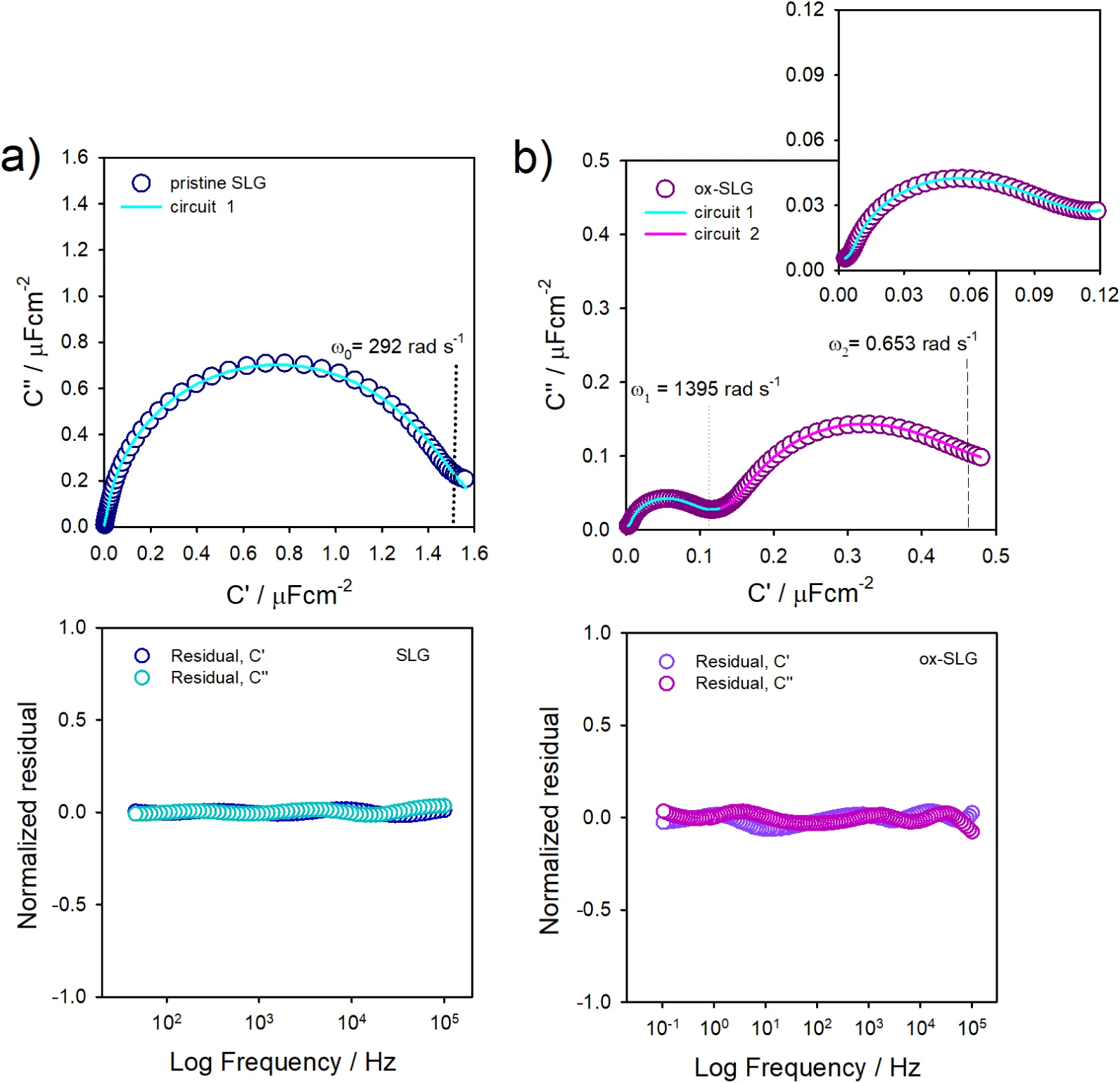
Capacitance Nyquist plots obtained by EIS for both (a) pristine and (b) oxidized graphene in the ionic liquid [BMIM]­BF_4_. The circular symbols represent the experimental data, while the solid lines correspond to the fitted curves based on the equivalent circuit illustrated in Figure 5. For ox-SLG, the cyan line (circuit 1) is the response of the branch fitted to pristine SLG (*R*_qt_, *C*_q_) and the magenta line (circuit 2) corresponds to the two additional branches associated with the oxygenated defect states ( $R_{qt_{1}}$ , $C_{q_{1}}$ and $R_{qt_{2}}$ , $C_{q_{2}}$ ); the colors match the two shaded blocks of Figure 5. The inset expands the high-frequency arc. The equilibrium frequencies (*ω*) associated with each relaxation response are marked by the vertical dashed lines for both graphene conditions. The lower panels show the normalized fitting residuals of *C*′ and *C*″ as a function of frequency, which are structureless across the measured frequency window.

As mentioned earlier, this semicircular response comes from an electronic relaxation process given by eq 6, where the relaxation time *τ* is the product of quantum capacitance *C*_q_ and quantum resistance *R*_q_, that is, *τ* = *R*_q_ *C*_q_. The quantum rate is the inverse, *ν* = 1/*τ*, and describes the speed of this process. As a result, the single C*­(*ω*) response seen for pristine SLG in Figure 4 *a* represents a distinct quantum RC (resistor–capacitor) process that can be modeled using simple circuit elements with quantum coherence. As shown before,^23,24^ fitting these data to a quantum RC circuit model based on eq 6 highlights the quantum nature of graphene’s capacitive response, provided that *R*_q_ is the expected universal constant.

This circuit, shown in the blue box in Figure 5, uses a resistance *R*_s_ that combines both contact and electrolyte resistances. It is connected in series with another resistance and a quantum capacitance, named *R*_qt_ and *C*_q_, respectively. The model also includes *C*_∞_. Since *C*_∞_ is much smaller than *C*_q_, the resistance *R*_qt_ adds to *R*_s_. Together, these constitute the quantum resistance, $R_{q}=2\pi \left(R_{s}+R_{qt}\right)$ . The 2*π* factor comes from the resonance condition *ωτ* = 1 (see eq 6). The experimental data fit the model well, as shown by the light blue line and a low chi-squared value (*χ* ^2^ = 0.056) in Table 1. The quantum resistance *R*_*q*_ is about 12.83 ± 0.23 kΩ, close to the expected value *R*_q_ = *R*_K_/2 = *h*/2*e* ^2^ ≈ 12.91 kΩ, with *R*_K_ the von Klitzing constant.^71^ We emphasize that this fit was performed with all parameters free; the agreement of the unconstrained *R_q_* with the quantized value is therefore an experimental result, not an imposed condition. This shows that the measured capacitance for pristine SLG in Figure 4 *a* agrees with the quantum electrodynamics of graphene.

**Figure 5:**
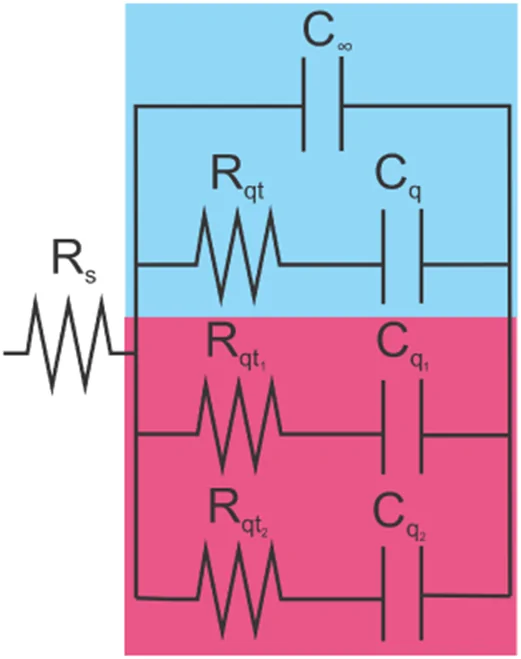
A quantum resistance-capacitance (RC) circuit model is used to fit electrochemical capacitance data obtained for both pristine and oxidized graphene surfaces. In this model, *R*_s_ represents all resistance phenomena associated with the electrical contact between the graphene surface and the electrolyte, while *C*_∞_ denotes the double electrical layer capacitance. Each parallel RC branch corresponds to a distinct relaxation process characterized by a time constant *τ* = *RC*, as defined in eq 6. The branch containing *R*_qt_ and *C*_q_ (fitted for pristine SLG) is assigned to the relaxation of the pristine graphene domains, while the two additional branches ( $R_{qt_{1}}$ , $C_{q_{1}}$ and $R_{qt_{2}}$ , $C_{q_{2}}$ , present only for ox-SLG) are assigned to the two oxygenated defect-state relaxations; the two shaded blocks are color-matched to the corresponding fitted curves of Figure 4b: cyan for circuit 1 and magenta for circuit 2.

**Table 1:** Values of the Circuit Parameters That Were Derived from the Fitting of the Complex Capacitance Response Measured for the Pristine SLG Using the Circuit Model Illustrated in the Blue Box in Figure 5 ^a^

Parameter	Value
*R*_s_	0.489 ± 0.065 kΩ
*C*_∞_	0.073 ± 0.002 nFcm^–2^
*R*_qt_	1.578 ± 0.567 kΩ
*C*_q_	1.435 ± 0.156 μFcm^–2^

a The standard error of each parameter was calculated from three independent replicates (separately fabricated and measured graphene devices; see the Electrochemical Measurements section).

The results obtained for pristine SLG from the quantum RC circuit analysis, in conjunction with the premises of QR theory, suggest that this approach can also be applied to the electronic structure and electrodynamics of more complex graphene-based systems such as oxidized SLG sheets. These oxidized structures were produced by electrochemical oxidation of pristine SLG. As discussed in the Graphene and Oxidized Graphene Structures section, the oxidation process disrupts the hexagonal carbon lattice, resulting in the incorporation of oxygen-containing moieties on the SLG surface. Thus, electrochemically oxidized SLG (ox-SLG) can be structurally described as a composite of pristine graphene domains and regions functionalized with oxygenated groups.^29,30^

Figure 4 *b* presents the Nyquist capacitive plot (*C*″ vs. *C*′) for ox-SLG at the OCP of the interface (−0.434 ± 0.065 V) in [BMIM]­BF_4_. In this ionic liquid, the capacitive response displays an additional relaxation process, seen as a new semicircle at lower frequencies in the Nyquist plot, in addition to the higher-frequency semicircle observed for pristine SLG. Building on this observation, the quantum RC circuit analysis considers each relaxation process as corresponding to a distinct RC quantum state associated with the electronic structure of ox-SLG. Fitting the higher-frequency semicircular response with the circuit shown in the blue box (*R*_s_, *C*_∞_, *R*_qt_, and *C*_q_) achieves excellent agreement with the experimental data, with a chi-squared (*χ* ^2^) value of 0.023.

The parameters extracted from this fitting are summarized in Table 2. Using the relation *R*_q_ = 2*π*(*R*_s_ + *R*_qt_), a quantum resistance value of *R*_q_ = 12.75 ± 0.3 kΩ was obtained. These results demonstrated that the first relaxation process, identified at higher frequencies in the complex capacitance spectrum of ox-SLG, corresponds to the same electronic states represented by the single semicircular response recorded for pristine SLG. Although the value of *C*_q_ decreased from 1.43 to 0.12 μF cm^–2^ due to oxidation-induced modifications in the electronic DOS, the quantized nature of *R*_q_ was preserved in both cases. This finding indicates that the capacitive response arises from the pristine graphene regions that remain after electrochemical oxidation, as expected.

**Table 2:** Circuit Parameter Values Were Obtained by Fitting the Complex Capacitance Response Measured for the Oxidized SLG (ox-SLG) Using the Circuit Model Illustrated in Figure 5 ^a^

Parameter	Value
*R*_s_	0.549 ± 0.076 kΩ
*C*_∞_	0.020 ± 0.001 *μ*F cm^–2^
*R*_qt_	1.473 ± 0.345 kΩ
*C*_q_	0.122 ± 0.006 *μ*F cm^–2^
$R_{qt_{1}}$	1.508 ± 0.267 kΩ
$C_{q_{1}}$	0.148 ± 0.004 *μ*F cm^–2^
$R_{qt_{2}}$	1.505 ± 0.471 kΩ
$C_{q_{2}}$	0.205 ± 0.007 μF cm^–2^

a The standard error for each parameter was computed from three independent replicates (separately fabricated, oxidized, and measured graphene devices; see the Electrochemical Measurements section).

Building upon a previous quantum RC circuit analysis of a different graphene-derived structure—a reduced graphene oxide (rGO) nanofilm electronically coupled to a conducting electrode via a molecular monolayer, which also exhibited a two-semicircle capacitive response^47^—the present analysis investigates the additional, or second, relaxation process observed at lower frequencies for ox-SLG in Figure 4. This investigation began by assuming that the complex capacitive response results from one or more electronic states linked to oxidized defects within the SLG sheet. To characterize this capacitive behavior using quantum RC dynamics, the principles of the QR theory were applied. According to QR theory, when electronic states in 0D, 1D, and 2D structures encounter a time-dependent electrical perturbation in an electrolytic medium, screening of the electric field by solution ions over these perturbed states enables coherent communication with them.^38,41^ This process leads to a relaxation resistance described by *R*_q_ = *h*/2*e* ^2^ ≈12.91 kΩ, as predicted by the Landauer–Büttiker electron transport formalism.

Under this physical assumption, the optimal circuit fitting for the second relaxation process was achieved by modeling the capacitive response as two additional quantum RC contributions. During the first stage, the parameters previously fitted for the initial capacitive relaxation at higher frequencies (using the circuit in the blue box) were kept fixed. Then, the second relaxation response—represented by the additional capacitive semicircle—was modeled by introducing two new RC branches in parallel, as illustrated in Figure 5. These branches, containing the elements $R_{qt_{1}}$ , $C_{q_{1}}$ , $R_{qt_{2}}$ , and $C_{q_{2}}$ , account for two distinct relaxation processes with characteristic times $\tau_{1}=R_{q_{1}}C_{q_{1}}$ and $\tau_{2}=R_{q_{2}}C_{q_{2}}$ , where $R_{q_{n}}=2\pi \left(R_{s}+R_{qt_{n}}\right)$ .

Because the two strongly overlapping semicircles of the low-frequency response cannot be uniquely deconvoluted with all four parameters of the two branches free—a well-known degeneracy of overlapping RC relaxations—the value of *R*_q_ established by the unconstrained fits of both pristine SLG (12.83 ± 0.23 kΩ) and the high-frequency ox-SLG relaxation (12.75 ± 0.30 kΩ) was imposed as a physical constraint, i.e., the values of $R_{qt_{1}}$ and $R_{qt_{2}}$ were constrained so that $R_{q_{1}}=R_{q_{2}}\approx 12.91\;k\Omega$ . The fitting software then refined the capacitances $C_{q_{1}}$ and $C_{q_{2}}$ , resulting in excellent agreement with the experimental data (*χ* ^2^ = 0.0160), as shown in Figure 4 *b*. We stress that this procedure constitutes a consistency test rather than a demonstration: the constrained model asks whether the low-frequency data are compatible with coherent quantum RC relaxation at the independently measured quantized resistance, and the answer is affirmative. A sensitivity analysis in which $R_{qt_{1}}$ and $R_{qt_{2}}$ were left free is reported in Section S6 of the Supporting Information; the free fit converges to $R_{q_{1}}=12.79\pm 0.24k\Omega$ and $R_{q_{2}}=12.87\pm 0.21k\Omega$ , statistically indistinguishable from *h*/2*e* ^2^, with no significant improvement in *χ* ^2^ (0.0155 against 0.0160 for the constrained fit, over the same 120 experimental frequency points), confirming that the constraint resolves a parameter degeneracy rather than forcing agreement. The resulting circuit parameters from this analysis of the complex capacitance response for ox-SLG are summarized in Table 2.

This fitting approach is based on physical constraints imposed during the circuit fitting procedure. Specifically, the resistance values $R_{qt_{1}}$ and $R_{qt_{2}}$ were set in accordance with the condition of coherent electronic communication (*R*_q_ = 12.91 kΩ, as independently measured by the unconstrained fits described above), while the quantum capacitance values $C_{q_{1}}$ and $C_{q_{2}}$ —or equivalently, the DOS, given that *C*_*q*_ ∝ DOS—were adjusted by the fitting software within this constraint. The adequacy of the selected equivalent circuit was further verified by comparison with plausible alternative models—a Randles-type circuit with a constant-phase element, a two-branch RC model without splitting of the low-frequency response, and a finite-length transmission-line model—none of which describes the data as well or provides a physically interpretable connection to the electronic DOS; the full comparison, including fitted parameters, *χ* ^2^ values, and residuals, is given in Section S6 of the Supporting Information. Moreover, the attribution of the low-frequency relaxation to the oxidized graphene itself, rather than to the electrolyte, the Si/SiO_2_ substrate, or the Ti/Au contacts, follows from the differential design of the experiment (Section S7 of the Supporting Information): the pristine-SLG measurement, performed in the same cell, with the same electrolyte batch, the same substrate, and the same contact architecture, constitutes the direct control for all of these extrinsic contributions and exhibits a single capacitive relaxation. Since oxidation is the only variable altered between the two measurements, the additional low-frequency relaxation can only originate from the oxidation-induced modification of the graphene electronic structure. Consistently, the amplitude of this relaxation correlates with the anodic charge passed during the oxidation protocol (Section S10 of the Supporting Information), and the narrow *I*_D_/*I*_G_ distribution across 12 Raman locations (Section S4 of the Supporting Information) rules out macroscopic surface heterogeneity as its origin. As a result, the second relaxation process observed for ox-SLG at lower frequencies corresponds to two quantum RC elements, as shown in Figure 5. These two superimposed electronic contributions—two overlapping capacitive semicircles—are linked to at least two oxygenated defect states whose electronic communication satisfies the principle of quantum coherence. Therefore, oxidizing SLG leads to the formation of two new defect-related electronic states in addition to those found in pristine graphene. However, assigning the specific oxygenated chemical defects responsible for these ECS-resolved states requires further analysis. By examining the equilibrium *C*_q_ contribution for each relaxation process, which directly reflects the electronic structure, each type of oxygenated defect can be identified at a specific electron energy, since *E* ∝ *e* ^2^/*C*_q_. This analysis uses the relationship *C*_q_ ∝ DOS and will be discussed in the following section.

### Electronic Density of States of Pristine and Oxidized SLG Structures

As described in the Electrochemical Measurements section, the electronic DOS, expressed as *C*_q_/*e* ^2^ or equivalently through the proportionality *C*_q_ ∝ DOS,^23,37^ can be determined from the Nyquist plots shown in Figure 4. This measurement is performed by applying a potential scan at the equilibrium *ω* frequency, defined as the frequency at which the capacitive semicircle closes in the low-frequency region of the spectrum, as highlighted in Figure 4 for both pristine and oxidized SLG. This frequency corresponds to the equilibrium quantum capacitance, *C*_q_, at which *E*_*μ*,0_ = *g*_e_ *n*_0_(*e* ^2^/*C*_q_) is obtained, representing the energy associated with the equilibrium electronic DOS of the SLG structures.

Figure 6 shows the experimentally resolved electronic DOS for both SLG conditions, measured at the equilibrium frequencies, *ω*, as indicated in Figure 4. For the pristine SLG structure, this frequency was denoted as *ω*_0_ = 292 rad s^–1^. A V-shaped DOS function—characteristic of the 2D structure of graphene^16,23^ —was measured, with a *C*_q_ minimum located at −0.78 ± 0.02 V. This minimum corresponds to the potential *E*_F_/*e* associated with the Fermi level, also referred to as the Dirac point of graphene. The V-shaped DOS follows a linear dispersion relation between energy and frequency, a hallmark of graphene’s electronic structure. This behavior is interpreted using Dirac’s equation (see eq 2), which predicts relativistic quantum electrodynamics in this type of structure. Specifically, it predicts non-dispersive, massless fermionic transport at the Dirac point.

**Figure 6:**
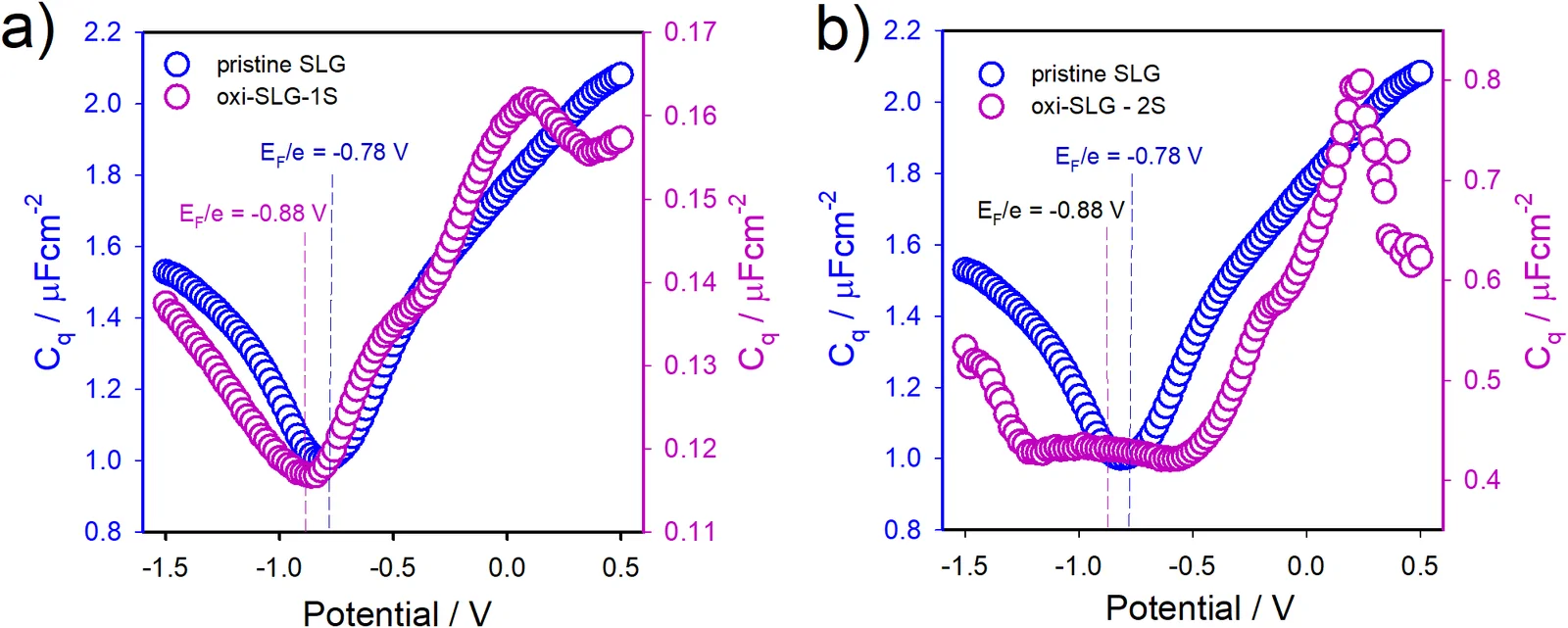
Responses of *C_q_* as a function of potential measured by EIS for both pristine and oxidized graphene surfaces. Due to the proportional relationship *C*_q_ ∝ *DOS*, these responses reflect the electronic density of states (DOS) of the graphene structures. (a) Comparison of the electronic DOS measured for pristine graphene at a constant frequency *ω*_0_ with that of oxidized graphene at a constant frequency *ω*_1_. (b) Comparison between the DOS of pristine graphene and that of oxidized graphene, measured at a constant frequency *ω*_2_. These frequencies are highlighted in Figure 4.

In an ideal, free-standing, and charge-neutral SLG sheet, the quantum *C*_q_ minimum theoretically occurs at 0 V on the absolute scale.^72,73^ However, in experimental electrochemical setups, the applied potential is referenced to an external electrode, which shifts the Dirac point voltage due to extrinsic effects.^17,23,24^ For instance, in the studied SLG samples, unintentional doping from the Si/SiO_2_ substrate, transfer residues, and adsorbed species shifts the Fermi level away from charge neutrality. The measured potential also includes work function differences and interfacial dipoles between graphene and the electrolyte/reference system.^17,72,74,75^ Thus, the *C*_q_ minimum is found at −0.78 V.

Two semicircle-shaped responses for ox-SLG appeared in the Nyquist capacitive plot, revealing two equilibrium frequencies (Figure 4 *b*) at *ω*_1_ = 1395 rad·s^–1^ and *ω*_2_ = 0.653 rad·s^–1^. QRS then measured two electronic contributions for ox-SLG, associated with *ω*_1_ and *ω*_2_. Figure 6 *a* shows a comparison between the DOS obtained for pristine SLG, measured at *ω*_0_, and that for the ox-SLG structure at *ω*_1_. The latter corresponds to the first capacitive semicircle or relaxation identified at higher frequencies. As illustrated in Figure 6 *a*, the values of *C*_q_ measured for ox-SLG are about an order of magnitude lower than those for pristine SLG. Oxidation creates many defects and adds oxygen groups. These defects disrupt the C–C bonds, causing carrier localization and lowering the DOS near the Dirac point.^72,76,77^ Since *C*_q_ depends on the DOS, less DOS from oxidation means lower *C*_q_ for ox-SLG. Thus, the smaller quantum capacitance for ox-SLG in Figure 6 *a* follows this logic and matches the expected *C*_q_ response, as seen in the Nyquist capacitive analysis in Figure 4.

A qualitative comparison, apart from the magnitude difference between SLG and ox-SLG, reveals further oxidation effects on SLG’s electronic structure. Oxidized SLG shows a distorted, asymmetric V-shaped profile between the inflection (Dirac) points, indicating the loss of the massless-fermion quantum electrodynamics of pristine SLG due to oxidation. The shift of the *C*_q_ minimum (Fermi level) to more negative potentials, from −0.78 to −0.88 V, further confirms oxidation by indicating p-type doping—meaning holes are injected (or electrons are ejected) into the graphene lattice, a clear sign of oxidation. The electronic DOS, derived from the first semicircular capacitive contribution at higher frequencies (see Figure 4 *b*), retains a V-shaped profile, supporting the view that the capacitive contribution (or electron quantum RC relaxation) arises from residual pristine graphene that persists after oxidation, aligning with the Raman and XPS analyses.

By analyzing the response of *C*_q_ at *ω*_2_ = 0.653 rad·s^–1^, which is associated with the second capacitive contribution observed at lower frequencies in Figure 4 *b*, a distinct behavior was identified. Figure 6 *b* compares this DOS with that of pristine SLG, revealing the loss of the characteristic V-shaped profile and the emergence of a maximum, rather than a minimum, of *C*_q_ at −0.88 V. This DOS feature at the Fermi level is attributed to the presence of a distribution of oxygenated defect states that modify the electronic structure at this energy level. Since this DOS, recorded at the frequency *ω*_2_, corresponds to the analysis of the second capacitive contribution in Figure 4 *b*, it can be inferred that this contribution originates from defect states associated with oxygen-containing functional groups within the SLG structure.

The analysis of the DOS conducted in this section, together with the discussion of quantum RC circuits from the preceding section, demonstrates the potential of the QRS approach for characterizing the electronic contributions of both pristine and oxidized SLG structures of graphene. Building on this foundation, the next section advances the analysis by exploring how evaluating the energy associated with each type of quantum RC relaxation enables the identification of defects introduced into SLG via electrochemical oxidation.

### Energy-State and Spectroscopic Analysis

The QR framework, as discussed in the Quantum-Rate Theory and Quantum-Rate Spectroscopy sections, establishes that *ν* = *g*_s_ *g*_e_ *e* ^2^/*hC*_q_, consistent with quantum coherence. Accordingly, the electrochemical energy can be written as *E*_*μ*_ = *hν* = *g*_s_ *g*_e_ *e* ^2^/*C*_q_. Assuming *g*_s_ = *g*_e_ = 2, this leads to *E*_*μ*_ = 4*e* ^2^/*C*_q_. This expression defines an energy state that reflects the system’s electronic structure. Notably, this state relies solely on the experimentally accessible *C*_q_, which can be measured directly through EIS, as previously demonstrated. Thus, the energy function *E*_*μ*_ can be readily determined from the *C*_q_ response at various potentials. In summary, *E*_*μ*_ for both pristine and oxidized SLG can be derived from their electronic density of states, as presented in Figure 6. For clarity, the complete conversion chain employed throughout this work is Z*­(*ω*) → C*­(*ω*) = 1/jωZ*­(*ω*) → *C*_q_(*V*) (taken at the equilibrium frequency, where the capacitive semicircle closes) → DOS­(*V*) = *C*_q_/*e* ^2^ (in J^–1^ cm^–2^, equivalently *C*_q_/*e* in eV^–1^ cm^–2^ on the electronvolt energy axis used here; or, after normalization by the number of probed states $N=\left(1/e\right)\int_{V_{1}}^{V_{2}}C_{q}dV$ with *V*_1_ = −1.5 V and *V*_2_ = 0.5 V, as the normalized per-state DOS in eV^–1^, which is the quantity compared with the per-atom DFT DOS) → *E*_*μ*_(*V*) = *g*_s_ *g*_e_ *Ne* ^2^/*C*_q_. The full derivation, including units at each step, the sensitivity of the results to the integration limits, and the propagation of the *C*_q_ fitting uncertainties (Tables 1 and 2) and of the potential-axis uncertainty (reference-electrode calibration and drift; Electrochemical Measurements section) into the DOS and *E*_*μ*_ functions, is presented in Section S8 of the Supporting Information.

Because *C*_q_ represents the total capacitance arising from *N* quantum capacitors, each with individual capacitance *C*_q,i_ (i.e., *C*_q_ = *NC*_q,i_), the energy of a single state is obtained by incorporating the total number of states *N*, leading to *E*_*μ*_ = 4*N*(*e* ^2^/*C*_q_) = 4*e* ^2^/*C*_q,i_. The total number of quantum states *N* for pristine and oxidized SLG was calculated by integrating the corresponding *C*_q_ responses shown in Figure 6, using the relation $N=1/e\int_{V_{1}}^{V_{2}}C_{q}dV$ . The resulting values are summarized in Table S1 of the Supporting Information.

Therefore, using the relation *E*_*μ*_ = 4*Ne* ^2^/*C*_q_ and the values computed for *N*, the corresponding energy functions *E*_*μ*_ for the pristine and oxidized SLG structures were obtained, as depicted in Figure 7. This energy plot for SLG was made by resolving the electron wave-vector deviation *δk* around the *K* point using the dispersion relation in eq 2, which is derived within the low-energy Dirac approximation and is valid only for states near *K*. Accordingly, the experimental curves in Figure 7 are presented as effective near-*K* dispersions reconstructed from the measured *C*_q_(*V*) response, plotted over the reduced *k*-space region actually probed by the measurement (small *δk* around *K*, i.e., states near the Fermi level);^17,78,79^ they do not represent, and should not be read as, a band structure over the full Brillouin zone. The *k*-space reference positions used in this construction are located geometrically from the reciprocal lattice of graphene, taking the experimentally determined *K* (Dirac) point as the reference, following the symmetry-based construction detailed in the Supporting Information of ref 24; no tight-binding (or any other parametrized electronic-structure) calculation is involved, and the DFT/SIESTA calculations of the present work are used exclusively in the comparison of Figure 9.

**Figure 7:**
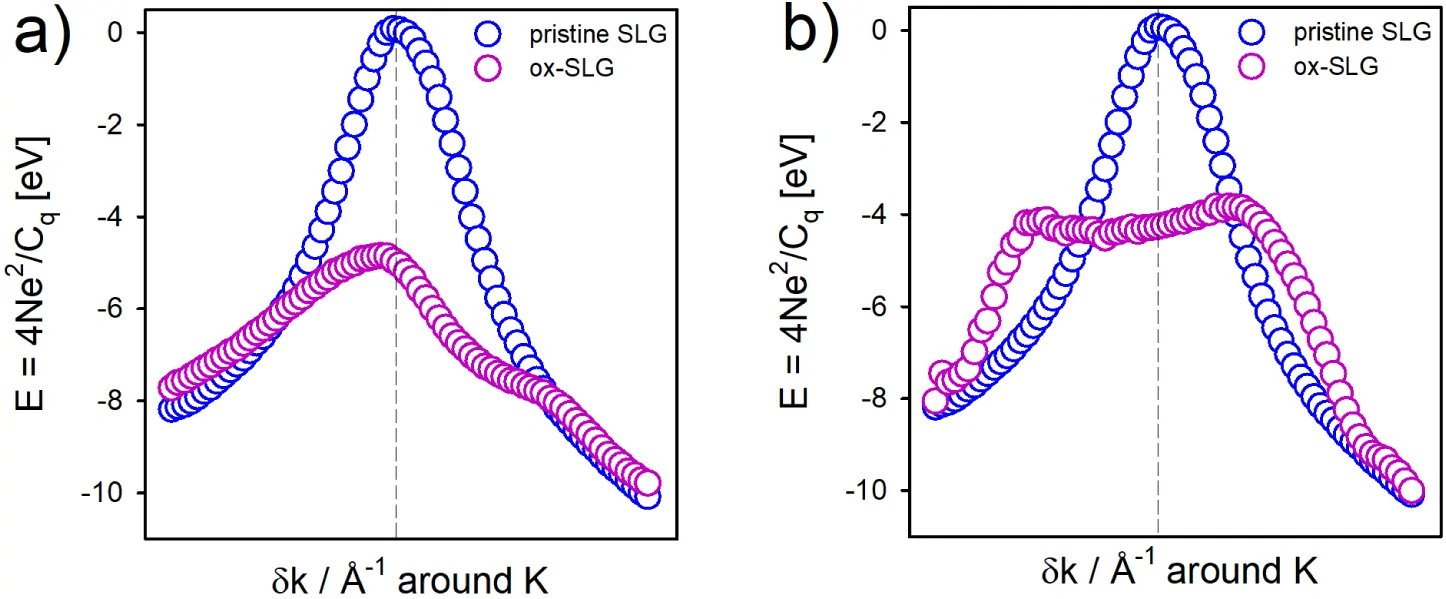
Energy functions were calculated for pristine and oxidized graphene structures based on their experimental electron DOS, as shown in Figure 6. The electronic energy was computed using *E_μ_* = 4*Ne* ^2^/*C*_q_ = 4*e* ^2^/*c*_q_, where *N* is the number of states or quantum channels and *c*_q_ is the quantum capacitance of a single capacitive state. (a) Energy functions for pristine and oxidized graphene at equilibrium frequency *ω*_1_. (b) Energy functions for both at equilibrium frequency *ω*_2_. The experimental curves are effective near-K dispersions reconstructed from *C*_q_(*V*) and are plotted against the reduced wave-vector deviation *δk* around the K point only; they do not represent the band structure over the full Brillouin zone. The *k*-space reference positions are located geometrically from the reciprocal lattice, using the experimental K (Dirac) point as reference (construction of ref 24).

For instance, in the case of the pristine SLG structure, *E*_*μ*_ exhibits a single maximum at the Dirac point, or *K* point, reflecting the characteristic electronic states of the pristine carbon framework. This *E*_*μ*_ response, typical of pristine SLG, has been obtained from measurements of its electronic structure using ARPES under high-vacuum conditions.^12,21^ Recent QRS measurements in an electrolytic medium at room temperature^24^ confirm these findings. Both ARPES and QRS methods show excellent qualitative and quantitative agreement. This demonstrates the capability of QRS to probe the electronic structure of graphene-based nanomaterials under ambient conditions using a conventional bench-top electrochemical setup, highlighting its potential as a widely accessible tool for advanced material characterization.

Consequently, two *E*_*μ*_ functions were computed for ox-SLG, matching the two DOS defect contributions measured under this condition (Figure 6), and each correlates with the equilibrium frequencies *ω*_1_ and *ω*_2_. To illustrate the differences, Figure 7 *a* presents a comparison of the *E*_*μ*_ function for pristine SLG and for ox-SLG at *ω*_1_. In this specific case, ox-SLG at *ω*_1_ retains the Dirac point maximum, as in pristine SLG, indicating the presence of pristine domains post-oxidation. Notably, ox-SLG at *ω*_1_ also shows additional maxima at about −0.33 and 0.34 V, attributed to defect states caused by oxygen-containing functional groups, which are absent in pristine SLG. In contrast, as shown in Figure 7 *b*, ox-SLG at *ω*_2_ is compared to pristine SLG. Here, it displays a minimum near the Fermi level and an additional feature at −0.04 V. The shift from a Dirac-point maximum to a minimum, or to a flattened, wider energy profile in ox-SLG at *ω*_2_, occurs due to defects from oxidation, linked to oxygen groups.^80,81^ This behavior does not mean electronic states are absent in this energy range. Collectively, these analyses demonstrate that QRS can resolve distinct electronic contributions from both pristine and oxidized domains within SLG structures.

To further clarify and identify the oxygenated groups associated with these distinct defect-related electronic contributions, a spectroscopic analysis was applied to the energy functions. Specifically, this analysis was based on subtracting the energy functions computed for both pristine and oxidized SLG, expressed respectively as *E_μ_*,_p_/*N*_p_ = 4*e* ^2^/*C*_q,p_ and *E*_*μ*,ox_/*N*_ox_ = 4*e* ^2^/*C*_q,ox_. In these expressions, *E*_*μ*,ox_/*N*_ox_ and *E*_*μ*,p_/*N*_p_ represent the total (per-state) energy functions, *N*_p_ and *N*_ox_ denote the number of states, and *C*_q,p_ and *C*_q,ox_ correspond to the total quantum capacitances, considering *N* quantum capacitors. Importantly, since *E*_*μ*,ox_/*N*_ox_ > *E*_*μ*,p_/*N*_p_ (that is, *C*_q,p_ > *C*_q,ox_), the energy difference Δ*E* can be expressed as Δ*E* = *E*_*μ*,ox_/*N*_ox_ – *E*_*μ*,p_/*N*_p_ = 4*e* ^2^/*C*_q,ox_ – 4*e* ^2^/*C*_q,p_. This distinction allows resolution of the energy function associated exclusively with the defect states, *E*_*μ*,ox_, as follows:

7 $$E_{\mu ,ox}=N_{ox}\Delta E+\frac{N_{ox}}{N_{p}}E_{\mu ,p}$$

Applying eq 7 to the ox-SLG energy function at equilibrium frequency *ω*_2_ (Figure 7 *b*), then baseline-correcting and spectrally deconvoluting the resulting differences, enables the identification of distinct energy functions for oxidized samples. This approach targeted the energy function, as it matches the second capacitive response at lower frequencies in Figure 4 *b*, which is associated with energy levels from oxygenated defect states. Thus, the spectroscopic analysis yielded an energy function exclusive to defect states. The subtraction analyses in Figure 8 showed three electronic contributions (energy maxima). The QRS and XPS data were then correlated and compared using these energy scales to characterize the respective chemical defects (Figure 3 *c*). This method allowed the assignment of specific energy contributions to chemical groups previously identified in SLG.

**Figure 8:**
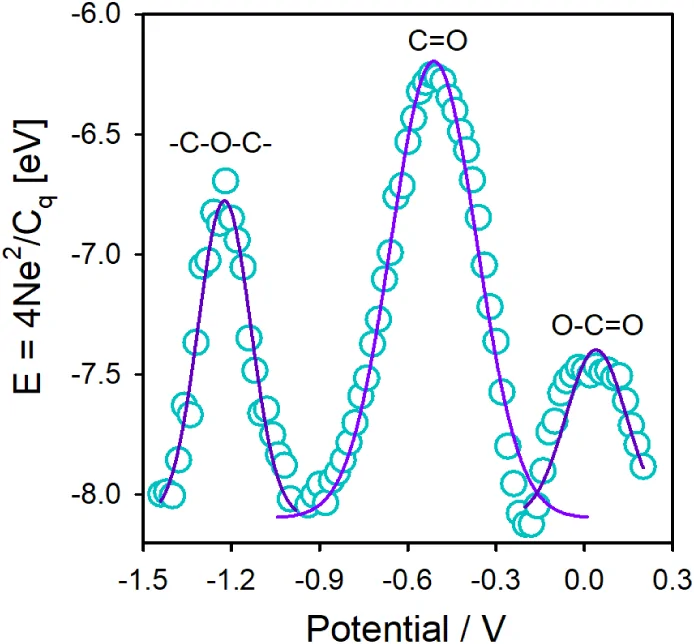
Subtracted energy function obtained through spectroscopic processing. Resulting function from subtraction of the energy functions shown in Figure 7b. As a result of the subtraction, distinct electronic states were resolved, which were correlated with the contributions identified in the XPS spectrum.

The first contribution, observed between −1.5 V and −0.8 V, is associated with epoxy (−C–O–C−) moieties introduced during the oxidation process. The second contribution appears in the range from −0.8 V to 0.2 V, corresponding to carbonyl (C=O) functionalities. Notably, the second range begins where the first ends, with no gap, indicating consecutive or potentially overlapping processes. The third contribution, recorded from −0.4 V to 0.2 V, is attributed to carboxylic (O–C=O) moieties, which overlap with the upper part of the carbonyl contribution range. These findings confirm that, although the QRS-based assignments are consistent with those obtained from conventional XPS analysis, QRS provides independent and detailed information about the electronic structure modifications induced by the chemical oxidation of graphene. It should be noted that the decrease in the relative area fraction of carbonyl (C=O) and carboxyl (O–C=O) groups observed by XPS (Table S3 of the Supporting Information) should not be interpreted as a proportional reduction in the contribution of these species to the states resolved by QRS. Whereas XPS quantifies the relative abundance of chemical bonds from the integrated area of each component of the C 1s spectrum, QRS is specifically sensitive to electronically active states near the Fermi level. Consequently, the detectability of a given chemical species by QRS depends on its local electronic activity rather than on its area fraction in the XPS spectrum. Thus, even a minority fraction of carbonyl or carboxyl groups remaining after oxidation may continue to generate a significant QRS signal, provided that these groups retain electronically active states near the Fermi level. This distinction accounts for why the two techniques, although complementary, do not necessarily yield quantitatively proportional results.

The effective energy resolution of QRS can be estimated from its instrumental contributions: the 10 mV RMS perturbation amplitude (∼28 meV peak-to-peak), thermal broadening of the Fermi–Dirac occupation at 298 K (≈3.5 *k*_B_ *T* ≈90 meV), the potential step of the sweep (20 mV), and the potential-axis uncertainty bounded by the OCP reproducibility across replicates (±21 mV for pristine and ±65 mV for oxidized SLG). Combined in quadrature, these yield an energy resolution of approximately 0.10–0.12 eV (see Section S8 of the Supporting Information), which is comparable to the peak-position precision of the XPS analysis (±0.1 eV), although the XPS spectral linewidths (FWHM of 0.8–1.8 eV) are dominated by chemical and lifetime broadening and are therefore not directly commensurable quantities. Importantly, QRS achieves this under ambient conditions, without the need for low temperatures, high-vacuum environments, or high-energy excitation sources typically required in electronic spectroscopic techniques. This represents a significant advantage, particularly for *in situ* and *in operando* studies. Hence, these results further demonstrate the reliability and consistency of the QRS method when employing EIS-derived experimental data.

Besides identifying individual electronic states, another important application of QRS is the exploration of oxidation levels. This can be accomplished by analyzing the correlation between QR spectra and computational chemistry calculations, as discussed below.

### Comparison with Computational Chemistry Simulations

This section demonstrates that the electronic DOS obtained for ox-SLG by using the QRS approach can reliably indicate the degree of oxidation. This is shown by comparing these results with DOS profiles calculated under various oxidation conditions using DFT, as outlined in the Computational Chemistry Simulations Section. In this analysis, three distinct oxidation levels were considered in the DFT calculations: low (1% oxidation of the graphene sheet), medium (5%), and high (10%). Building on this, the individual and combined effects of specific oxygen-containing functional groups on the electronic DOS of SLG were also investigated, focusing on epoxy, hydroxyl, and carboxylic groups.

Figure 9 shows a direct comparison between the electronic DOS profiles measured for ox-SLG at the *ω*_1_ and *ω*_2_ frequencies (see Figure 6) and those calculated by DFT for low and medium degrees of oxidation with epoxy functional groups, demonstrating a significant correlation between the theoretical (DFT) calculations and experimental (QRS) measurements. For a valid comparison between theoretical and experimental DOS, both were normalized based on the number of states per atom or molecule involved in the energy analysis associated with *C*_q_. In detail, the experimental DOS normalization involved dividing the total measured energy by *N*, the total number of measured states, yielding DOS = *C*_q_/*Ne* ^2^ = *c*_q_/*e* ^2^. This approach yields a DOS plot that reflects the valence (or frontier orbital) electronic energy states per atom or molecule. This method is necessary because the experimental DOS, given by *C*_q_/*Ne* ^2^ = *c*_q_/*e* ^2^, specifically measures the valence electronic states per atom or molecule, in line with the definition of *C*_q_.^38,82^ The *C*_q_ value only includes valence states that are accessible during the low-energy perturbation, as required by the QR premises and the definition of *C*_q_.

**Figure 9:**
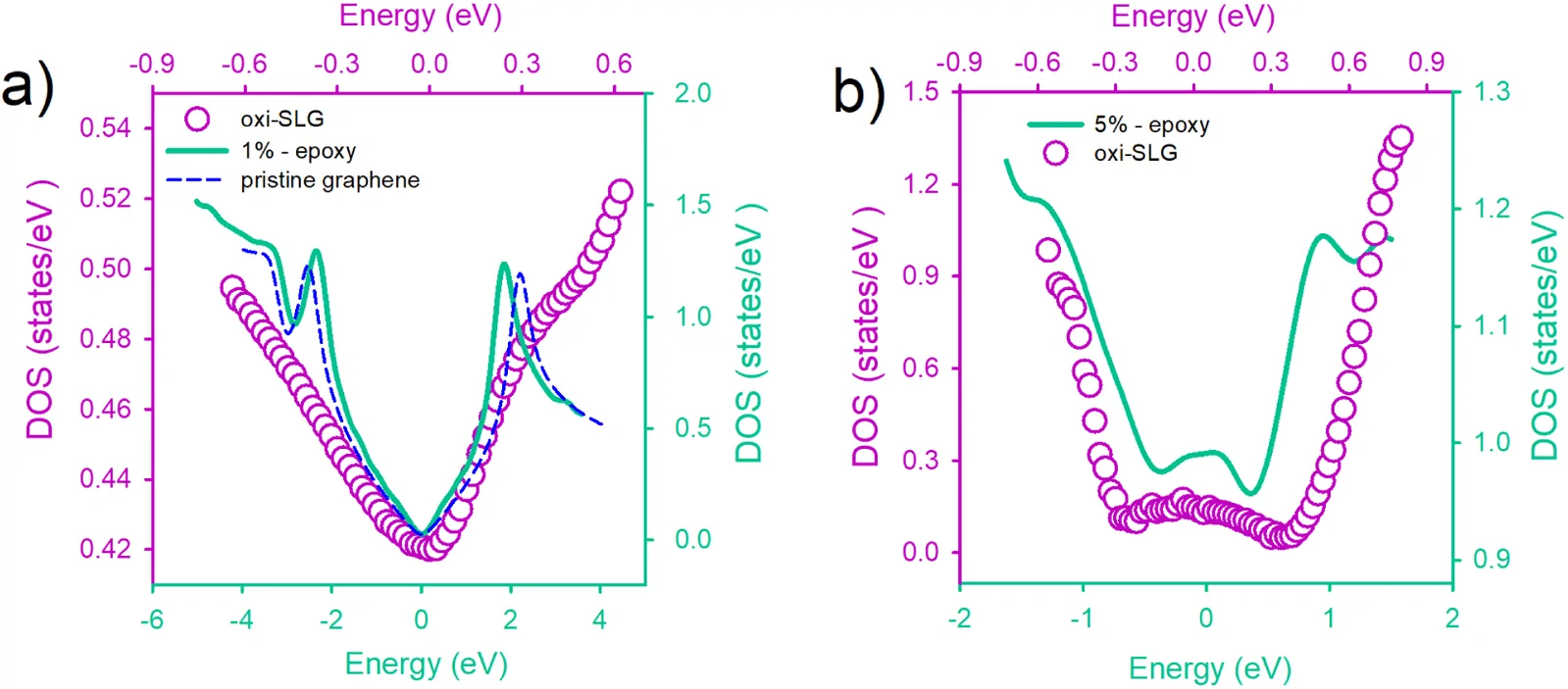
Comparison between the electronic DOS measured by EIS in [BMIM]­BF_4_ and those computationally simulated using DFT for oxidized graphene surfaces. (a) DOS measured at the constant equilibrium frequency *ω*_1_, compared with the simulated DOS for a graphene monolayer with 1% oxidation by epoxy groups. (b) DOS experimentally recorded at the constant frequency *ω*_2_, compared with the simulated DOS for a graphene structure with 5% oxidation by epoxy groups.

We normalized the DOS by *n* (per number of atoms involved in the simulation), as described in the Computational Chemistry Simulations section, to allow comparison with the DOS calculated via DFT. Figure 9 *a* shows that the DOS measured for *ω*_1_ (see Figure 6 *a*) qualitatively agrees with the DOS calculated for 1% oxidation with epoxy groups. For this oxidation level, the V-shaped DOS profile remains, indicating that the pristine electronic structure of SLG still reflects the strong influence of remaining carbon bonds despite oxidation. This observation supports our earlier analysis (see previous sections), which proposed that the DOS measured at *ω*_1_ in Figure 6 *a* results from the preservation of pristine carbon domains after oxidation. The differences between the DOS minima in Figure 9 *a* arise from the distinct nature of the simulated graphene model and the experimental setting. The nearly zero DOS minimum for the 1% epoxy calculation reflects an ideal, periodic, free-standing graphene lattice with low, uniform epoxy concentration, where the pristine electronic structure is largely preserved. In contrast, the ox-SLG sheet, supported on a Si/SiO_2_ substrate, is a disordered system with non-uniform oxygen functionalities, defects, and charge inhomogeneities. These factors introduce localized states near the Dirac point and prevent the DOS from vanishing.^13,17^ Despite differences in absolute DOS values, the similar overall shapes and trends of the calculated and experimental curves indicate that the simulations capture the qualitative features of graphene’s electronic structure upon oxidation.

Similarly, the measured electronic DOS profile of oxidized SLG at *ω*_2_ agrees with the simulated (DFT) DOS for a 5% oxidation level dominated by epoxy groups, as shown in Figure 9 *b*. The large DOS minimum obtained for the 5% epoxy calculation arises from the artificially high and perfectly periodic epoxy coverage imposed in the DFT supercell, which strongly breaks lattice symmetry and consequently overestimates the density of defect-induced states at the Fermi level.^72,73,83^ Experimentally, the ox-SLG DOS minimum is reduced by spatial averaging, thermal and electrostatic broadening, and substrate effects.^84^

It should be noted that the simulated and experimental DOS profiles do not match well under other oxidation conditions, such as varying amounts of epoxy groups or other oxygen-containing groups, as detailed in Section S2 of the Supporting Information. Therefore, it can be concluded that the experimental DOS observed at the equilibrium frequency *ω*_2_ is linked to states from oxygen groups on the SLG surface, with sp^3^-hybridized carbon bonds being the main chemical feature behind the electronic response. This conclusion aligns with the XPS analysis, which shows that electrochemical oxidation primarily forms epoxy groups on the SLG surface. Notably, XPS reveals a higher fraction of epoxy groups than of other functionalities. Furthermore, Figure 9 *b* supports this interpretation with a predicted epoxy content of about 5%. Comparatively, the structural models for 1% and 5% oxidized SLG, detailed in Section S3 of the Supporting Information, demonstrate that epoxy group coverage increases with oxidation, clarifying the differences between low (1%) and moderate (5%) oxidation levels.

## Conclusions

This work establishes QRS as a rigorous and practical spectroscopic tool for the *in situ* characterization of the electronic structure of oxidized single-layer graphene. The method operates at room temperature and ambient pressure using a portable, low-cost potentiostat—conditions that are inaccessible to high-vacuum techniques such as ARPES and XPS—and without the need for high-energy excitation sources. The defect-state energies resolved by QRS carry a precision comparable to the peak-position precision of XPS (±0.1 eV; see the resolution budget in the Energy State and Spectroscopic Analysis section) and are consistent with DFT calculations, demonstrating that the technique is not merely qualitative but quantitatively informative.

Through an integrated analysis combining Raman spectroscopy, XPS, and QRS, we unambiguously identify epoxy groups as the dominant oxidation-induced defect in electrochemically oxidized SLG, comprising approximately 5% of the carbon lattice. This assignment is independently corroborated by three orthogonal approaches: Raman spectroscopy identifies sp^3^-type structural disorder with a measured inter-defect distance of *L*_D_ = 31.58 ± 2.74 nm and a defect density of *n*_D_ ≈ (3.27 ± 0.67) × 10^10^ cm^–2^ (effective estimates within the assumptions of the Cançado point-defect model;^67^ note that *n*_D_ counts Raman-active defective domains rather than individual functional groups, as discussed in Section S5 of the Supporting Information); XPS resolves the chemical identity of oxygenated functional groups; and QRS directly maps the electronic DOS modifications associated with these defects under electrolyte conditions. The QRS-based spectral subtraction further resolves three distinct defect-state contributions—epoxy, carbonyl, and carboxylic—with energy ranges that are fully consistent with the XPS peak assignments.

The agreement between QRS-measured DOS profiles and DFT-simulated DOS for 1% and 5% epoxy oxidation validates the physical model underlying the QRS and demonstrates its ability to quantify oxidation levels. This makes QRS a uniquely positioned measurement technique: it delivers electronic structural information on spectroscopic quality under realistic, *in situ* conditions, using instrumentation that is orders of magnitude more accessible than ARPES or synchrotron-based methods. The approach introduced here is directly applicable to quality control of commercial graphene materials, *in operando* monitoring of graphene-based devices, and the broader characterization of 2D material surfaces, where defect-state engineering is central to performance.

## Supplementary Material



## Notes

Calculation of the number of electronic states from the *C*_q_ responses (Table S1); electronic density of states calculated by DFT for 1%, 5%, and 10% oxidation with epoxy and hydroxyl groups (Figure S1); optimized structural models for the 1% and 5% epoxy oxidation levels (Figure S2); Raman spectra recorded at 12 locations on ox-SLG and the corresponding *I*_D_/*I*_G_ statistics (Figures S3 and S4); estimation of the inter-defect distance and defect density from the Cançado relation; comparison of equivalent-circuit models, unconstrained sensitivity analysis of the low-frequency relaxation, and fitting residuals (Table S2, Figure S5); differential controls supporting the attribution of the low-frequency relaxation to the oxidized graphene; capacitance-to-DOS conversion chain, propagation of the fitting and potential-axis uncertainties, and the QRS energy-resolution budget; quantitative deconvolution of the C 1s XPS spectra of pristine and oxidized SLG (Table S3); electrochemical oxidation protocol, anodic charge passed by each device, and representative chronoamperograms (Table S4, Figure S6) (PDF)
