# Supplementary material for: In Situ Measurement of the Electronic Structure of Oxidized Graphene Employing Quantum-Rate Spectroscopy
Source: ACS Meas Sci Au. 2026 Oct 7;6(5):1769–85. doi: 10.1021/acsmeasuresciau.6c00179 (PMC13693214; doi:10.1021/acsmeasuresciau.6c00179)
Supplement: Supplementary file 1 [file tg6c00179_si_001.pdf]

Supporting Information: In Situ Measurement of the Electronic Structure of Oxidized Graphene Employing Quantum-Rate Spectroscopy

Edgar F. Pinzón,† Leandro Hostert,† Gabriela Dias da Silva,† and Paulo R. Bueno*,†,‡

†*Department of Physics and Mathematics, Institute of Chemistry, São Paulo State University (UNESP), Araraquara, 14800-060, São Paulo, Brazil*

‡*Centro Interdisciplinar de Química e Bioloxía (CICA) and Departamento de Química, Facultade de Ciencias, Universidade da Coruña, 15071 A Coruña, Galicia, Spain*

E-mail: paulo-roberto.bueno@unesp.br, paulo.bueno@udc.es

Phone: +55 (16) 3301-9642

S1 Calculation of the Number of Electronic States

Table S1: The total number of electronic states was computed for both pristine and oxidized graphene surfaces based on their C_q response.

Surface	Equilibrium frequency	N / states
pristine graphene	$\omega_0 = 292$ rad s^{-1}	7.49×10^{12}
oxidized graphene	$\omega_1 = 1395$ rad s^{-1}	6.07×10^{11}
oxidized graphene	$\omega_2 = 0.653$ rad s^{-1}	2.30×10^{12}

The total number of electronic states, N, associated with each graphene condition, pris-

tine and oxidized, was calculated using the definition of quantum capacitance, C_q, for a nanoscale structure, given by $C_q = e^2(dN/dE)$, where (dN/dE) represents the electronic density of states (DOS).[S1–S3] Starting from this relationship, N can be isolated as $N = 1/e \int_{V_1}^{V_2} C_q \, dV$, where e is the elementary charge, and the integral $\int_{V_1}^{V_2} C_q \, dV$ corresponds to the integrated response of C_q as a function of the applied potential. Thus, by first obtaining C_q as a function of V, it becomes possible to determine N via integration. In this analysis, the C_q responses shown in Figure 6 of the main text were integrated using the limits $V_1 = -1.5$ V and $V_2 = 0.5$ V. The resulting values of N for each C_q response are presented in Table S1, along with the equilibrium frequency at which each response was recorded. These calculated N values then enabled the determination of the electronic energy and DOS functions presented in Figures 7 and 9 of the main text, respectively.

S2 Calculation of Electronic Density of States by Density Functional Theory

The electronic density of states (DOS) was calculated for graphene with different levels of oxidation using density functional theory (DFT). This analysis examined the impact of adding epoxy and hydroxyl groups to the surface of graphene, as described in the Computational Chemistry Simulations section of the main text, on its properties. For each type of oxygen-containing group, three different levels were studied: 1%, 5%, and 10% oxidation. The electronic DOS was also calculated for a mixed case containing 5% epoxy and 5% hydroxyl groups (see Figure S1*b*). As shown in Figure S1, the DOS result for 5% epoxy oxidation matched best with the experimental data near the Fermi level, measured by QRS (see the Comparison with Computational Chemistry Simulations section of the main text). These findings suggest that the electrochemically oxidized graphene surface mostly has about 5% epoxy groups.

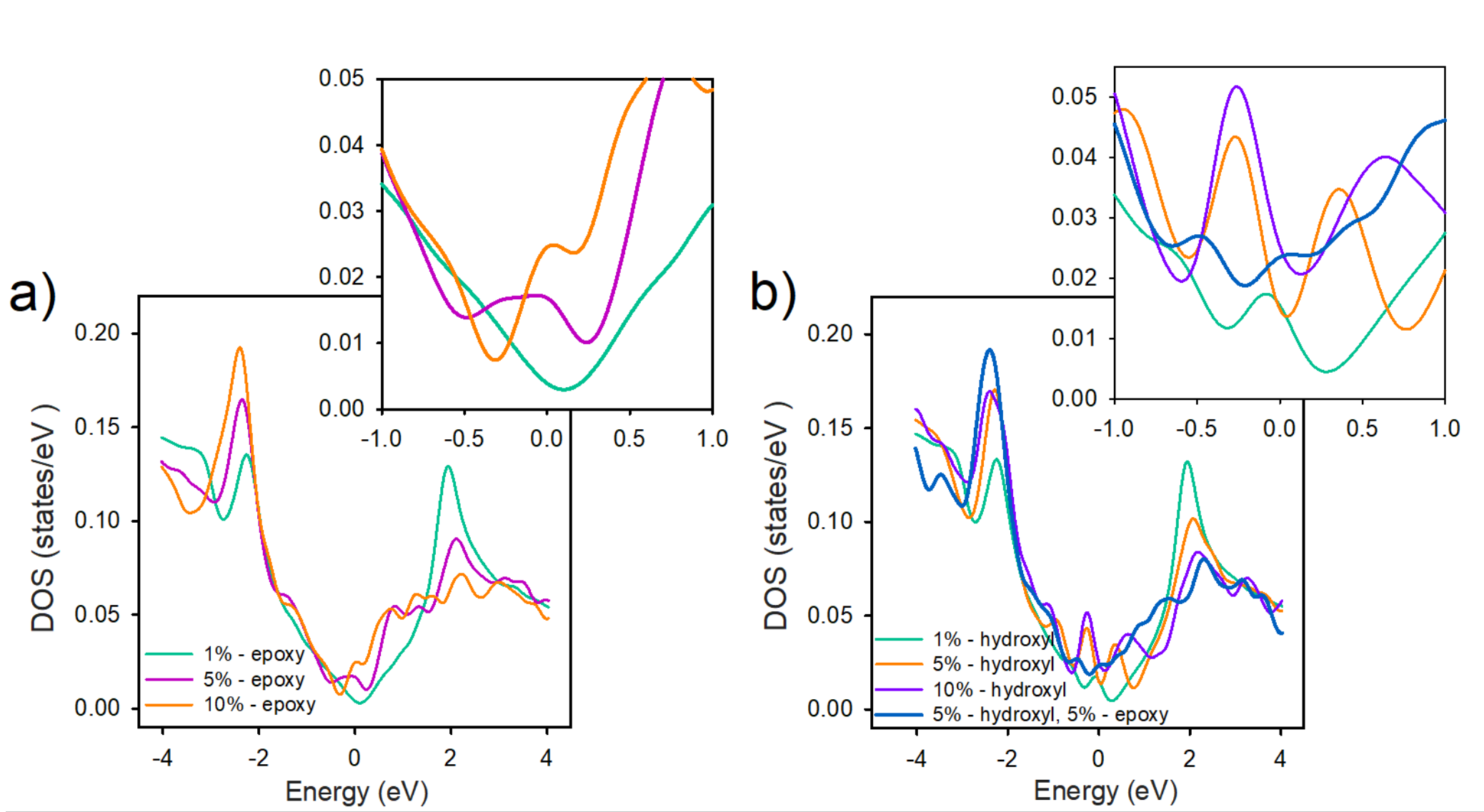

Figure S1: Electronic density of states (DOS) calculated via DFT for three distinct oxidation levels — low (1%), medium (5%), and high (10%) — with (a) epoxy and (b) hydroxyl functional groups. Panel (b) additionally shows the mixed case containing 5% hydroxyl and 5% epoxy groups. The insets in each plot provide a detailed view of the DOS near the Fermi level for each oxidation condition.

S3 Structural Models for the 1% and 5% Epoxy Oxidation Levels

All simulations used a single-layer graphene supercell with 180 carbon atoms. The 1% and 5%-epoxy models contained 2 and 9 epoxy (C–O–C) groups, respectively, each bridging two neighboring carbon atoms. Throughout this work the oxidation level denotes the oxygen-to-carbon atomic ratio of the supercell, so that 2, 9 and 18 oxygen atoms in 180 carbon atoms correspond to 1.1%, 5% and 10%, labelled for brevity as the 1%, 5% and 10% levels. All structures were fully geometry-optimized within the DFT framework. We note that one representative configuration of the epoxy groups was employed at each oxidation level; for each level, the functional groups were distributed as uniformly as possible over the graphene sheet, maximizing the distance between neighboring defects, a strategy adopted both to avoid artificial clustering and to minimize the artificial defect–defect interactions introduced by the

finite supercell. Other local arrangements are possible and may influence the quantitative results, but an exhaustive configurational sampling is computationally prohibitive, since the number of possible arrangements increases combinatorially with system size. Structural disorder is therefore not sampled by configuration averaging, and the calculated DOS should be read as a qualitative reference rather than a quantitative, disorder-averaged prediction. The specific features used for comparison with the QRS measurements are, however, robust to the configurational details: the preservation of the V-shaped DOS in the dilute limit and the emergence of oxygen-induced states near the Fermi level with increasing epoxy coverage are generic consequences of sp^3 functionalization of the graphene lattice, consistently reported across independent DFT studies employing different defect arrangements and supercells.[S4–S6] The fine structure of the defect peaks, which does depend on the specific arrangement, is not used in any quantitative comparison in this work. Figure S2 shows schematic representations of both oxidation conditions.

S4 Statistical Analysis of Raman Measurements for Oxidized SLG

Raman spectra were collected at twelve locations across the oxidized SLG (ox-SLG) surface to evaluate local structural variations (see Figure S3). All spectra exhibit well-defined D (∼1350 cm^{-1}), G (∼1580 cm^{-1}), and 2D (∼2630 cm^{-1}) bands, along with minor D′ and D + D″ contributions. Importantly, no significant peak shifts or systematic variations in relative intensities were observed, indicating a high degree of macroscopic structural uniformity. To ensure accuracy, the spectra were baseline-corrected and deconvoluted using Lorentzian profiles before calculating the I_D/I_G ratio. Furthermore, the D′ band (∼1620 cm^{-1}), which partially overlaps with the G band, was explicitly included in the fitting to avoid overestimating the G-band intensity. Finally, I_D and I_G values were extracted as the peak intensities of the fitted D and G bands, respectively.

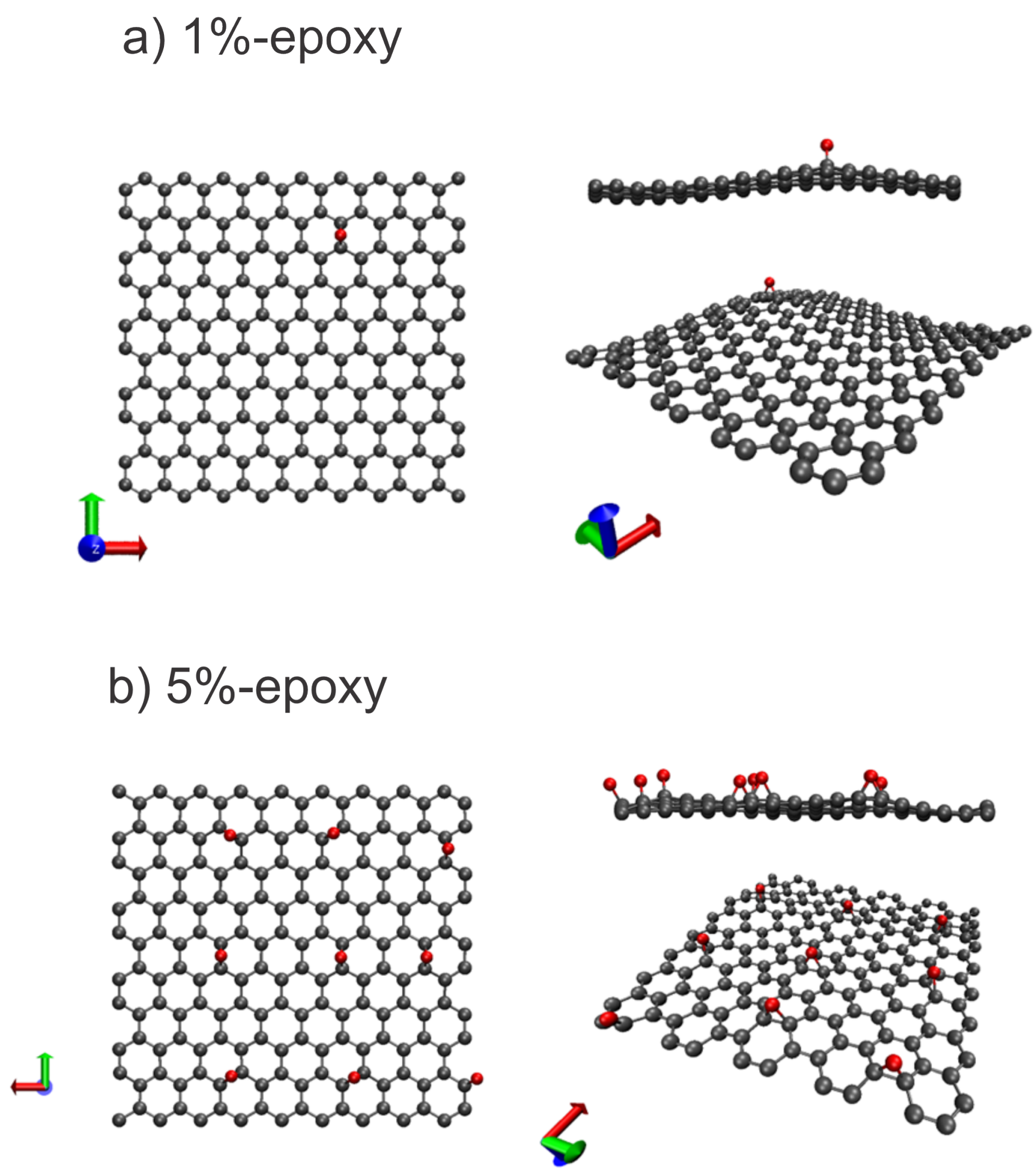

Figure S2: Optimized structures calculated using DFT for distinct oxidation levels with epoxy groups—low (1%) and medium (5%). Black and red spheres represent carbon and oxygen atoms, respectively. The panels are schematic and are intended to illustrate the epoxy bonding motif and the relative coverage of the two oxidation levels; they are not intended as an exact rendering of the number and positions of the functional groups in the supercells, which are specified in the text.

The box plot of Figure S4 shows a median I_D/I_G of ∼0.27–0.28 and an interquartile range of 0.26–0.30 (IQR ∼0.03–0.04), indicating limited variability across sampled regions. Whiskers extend from ∼0.24 to ∼0.43, with two outliers, at ∼0.23 and ∼0.46. Overall, the results indicate macroscopically consistent defect density, though the spread and the two outliers suggest some localized surface variations.

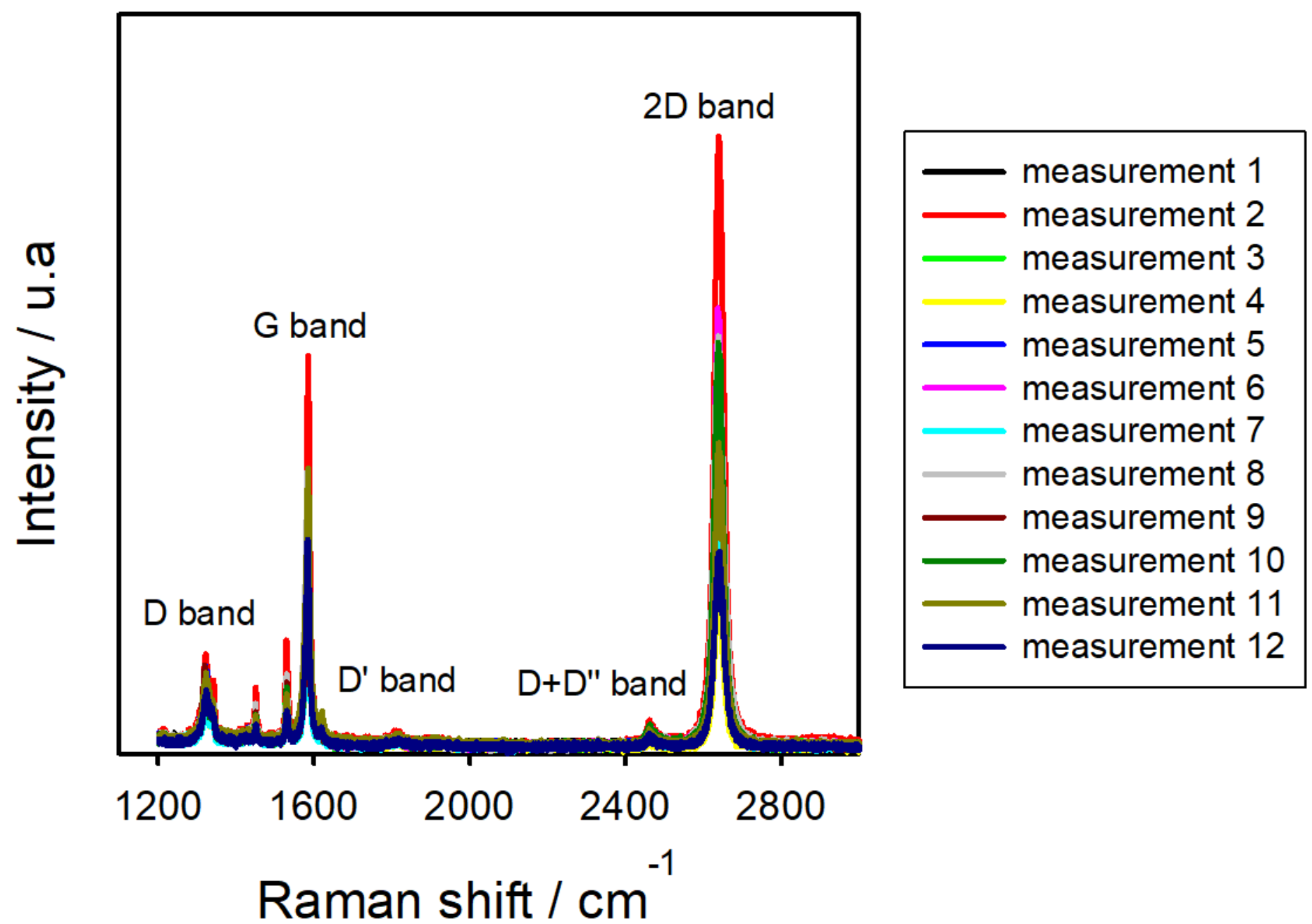

Figure S3: Raman spectra of oxidized SLG (ox-SLG) were collected at twelve different locations across the sample surface. All spectra exhibit a consistent profile with well-defined D, G, and 2D bands, along with minor D′ and D + D″ contributions, indicating high structural homogeneity and reproducibility.

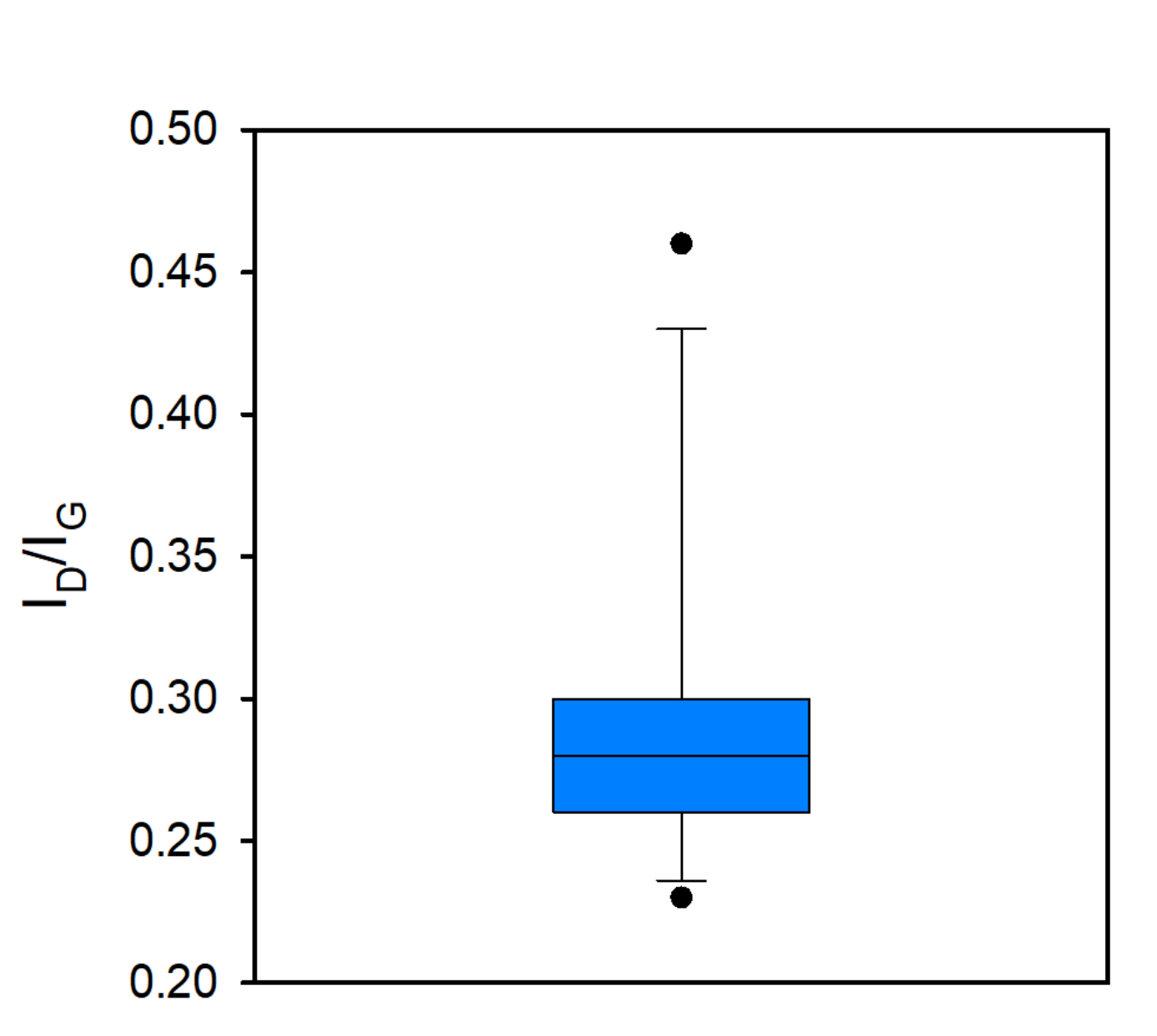

Figure S4: Boxplot of the I_D/I_G ratio from 12 independent measurements. The distribution shows a well-defined median and narrow interquartile range, indicating limited variability in defect density, with localized variations evidenced by the spread and two outliers.

S5 Estimation of the Inter-Defect Distance and Defect Density from Raman Spectroscopy

The defect density of ox-SLG was quantitatively evaluated from the I_D/I_G ratio in Raman spectroscopy. To obtain a physically meaningful parameter, the inter-defect distance (L_D) was calculated using the Cançado et al. model,[S7] which relates the I_D/I_G ratio to average defect spacing for a given excitation wavelength. In the low-defect-density regime, this relationship is expressed as:

$$\frac{I_D}{I_G} = C(\lambda)\,\frac{1}{L_D^2} \tag{S1}$$

where $C(\lambda)$ is a parameter that depends on wavelength. It is defined as $C(\lambda) = 1.8 \times 10^{-9}\lambda^4$, where λ is in nanometers. For the Raman measurements on ox-SLG samples, we used a He–Ne laser ($\lambda = 632.8\,\text{nm}$). In this case, the constant becomes $C(632.8\,\text{nm}) \approx 288$. Using the

I_D/I_G values obtained from the Raman spectra measured at twelve different localizations (Fig. S3), the average inter-defect distance was calculated to be $L_D = 31.58 \pm 2.74$ nm. This value shows that the system is in the low-defect-density regime, as defined by Ferrari & Robertson[S8] and quantified for graphene by Lucchese *et al.*[S9] The graphene lattice remains largely intact, with defects spatially isolated. To estimate defect density (n_D), we assumed a random distribution of point defects across the graphene surface. With this approximation, n_D is inversely proportional to each defect's associated area:

$$n_D = \frac{1}{\pi L_D^2} \tag{S2}$$

By substituting the L_D values obtained for the twelve localizations into the previous equation, the average defect density was calculated to be $n_D \approx (3.27 \pm 0.67) \times 10^{10}$ cm^{-2}. These values should be read as effective, order-of-magnitude estimates: the Cançado relation[S7] assumes point-like (0D) defects randomly distributed on the surface, the low-defect-density regime ($L_D \gtrsim 10$ nm, satisfied here), and calibration constants obtained for Ar^+-bombarded graphene, whereas chemical oxidation produces extended sp^3 patches whose Raman activation cross-section may differ from that of vacancies. The quoted uncertainties correspond to the standard deviation over the twelve independent locations (I_D/I_G: median 0.28, interquartile range 0.26–0.30, full range 0.23–0.46). This low n_D confirms that defects are sparsely distributed in the graphene lattice. Additionally, the inter-defect distance (∼32 nm) far exceeds the Raman relaxation length (∼2–3 nm),[S9] ensuring defect-induced scattering regions remain distinct. This supports the assumption of electronically isolated defects and validates the use of periodic supercell models in DFT calculations. Finally, we stress that n_D counts Raman-*active* defective domains rather than individual sp^3 sites: each activated region has a radius r_a of a few nanometres[S9] and may contain many oxygen-containing groups. The quantity to be compared with the ∼5% epoxy coverage independently inferred from the QRS/DFT analysis is therefore the areal fraction of the lattice contained within such domains, $\pi r_a^2 n_D \approx 1\%$ for $r_a \approx 3$ nm, which is of the same order of magnitude as

the QRS/DFT estimate — and not the ratio between n_D and the areal density of carbon atoms (3.8×10^{15} cm^{-2}), which would compare a density of defective domains with a density of individual functional groups. Agreement closer than one order of magnitude should not be expected here: r_a is calibrated for ion-bombarded graphene, and the local structure of oxidized graphenes varies substantially with the preparation route, so the two figures are best read as mutually compatible rather than as a quantitative match. Within this reading, Raman spectroscopy, DFT modelling and QRS are consistent with a sparsely distributed set of nanometre-sized, epoxy-rich sp^3 domains on an otherwise intact graphene lattice.

S6 Equivalent-Circuit Model Comparison and Unconstrained Sensitivity Analysis

To justify the equivalent circuit adopted in the main text (Figure 5), the complex-capacitance response of ox-SLG was additionally fitted with three plausible alternative models: (i) a Randles-type circuit in which the quantum capacitance is replaced by a constant-phase element (CPE), the standard empirical description of depressed semicircles; (ii) a two-branch RC model, i.e., one RC branch per observed semicircle, without splitting the low-frequency response into two quantum RC contributions; and (iii) a finite-length transmission-line (distributed) model, appropriate if the low-frequency relaxation arose from distributed ionic transport rather than discrete electronic states. Table S2 summarizes the number of free parameters and the χ^2 value of each fit, and Figure S5 the corresponding residuals. The model adopted in the main text is preferred on grounds of goodness of fit, structure-free residuals, physical interpretability (direct connection between each capacitance and the electronic DOS via $C_q = e^2(dn/dE)$), and parsimony, since fixing R_q at its independently measured quantized value leaves only two free capacitances for the low-frequency response.

In addition, a sensitivity analysis was performed in which the constraint on R_{qt_1} and R_{qt_2} was released, so that the four parameters of the low-frequency block (R_{qt_1}, C_{q_1}, R_{qt_2}, C_{q_2})

were fitted freely and the full circuit carried eight free parameters instead of six. The free fit converges to $R_{q_1} = 12.79 \pm 0.24$ kΩ and $R_{q_2} = 12.87 \pm 0.21$ kΩ, statistically indistinguishable from $h/2e^2 \approx 12.91$ kΩ, with $\chi^2 = 0.0155$, i.e., no statistically significant improvement over the constrained model ($\chi^2 = 0.0160$) despite the two additional adjustable parameters, both fits being performed over the same 120 experimental frequency points. This confirms that the constraint applied in the main text resolves a parameter degeneracy of the two overlapping semicircles rather than forcing the data into agreement with the quantized value.

Table S2: Comparison of equivalent-circuit models fitted to the ox-SLG complex-capacitance data. The number of free parameters refers to the complete circuit; all fits were performed over the same 120 experimental frequency points.

Model	Free parameters	χ^2	Comment
Quantum RC (main text, constrained)	6	0.0160	DOS-interpretable
Quantum RC (unconstrained)	8	0.0155	degenerate parameters
Randles + CPE	4	0.119	$n = 0.80 < 1$, non-physical
Two-branch RC	6	0.153	misses the low-frequency splitting
Transmission line	5	0.253	no 45° regime observed

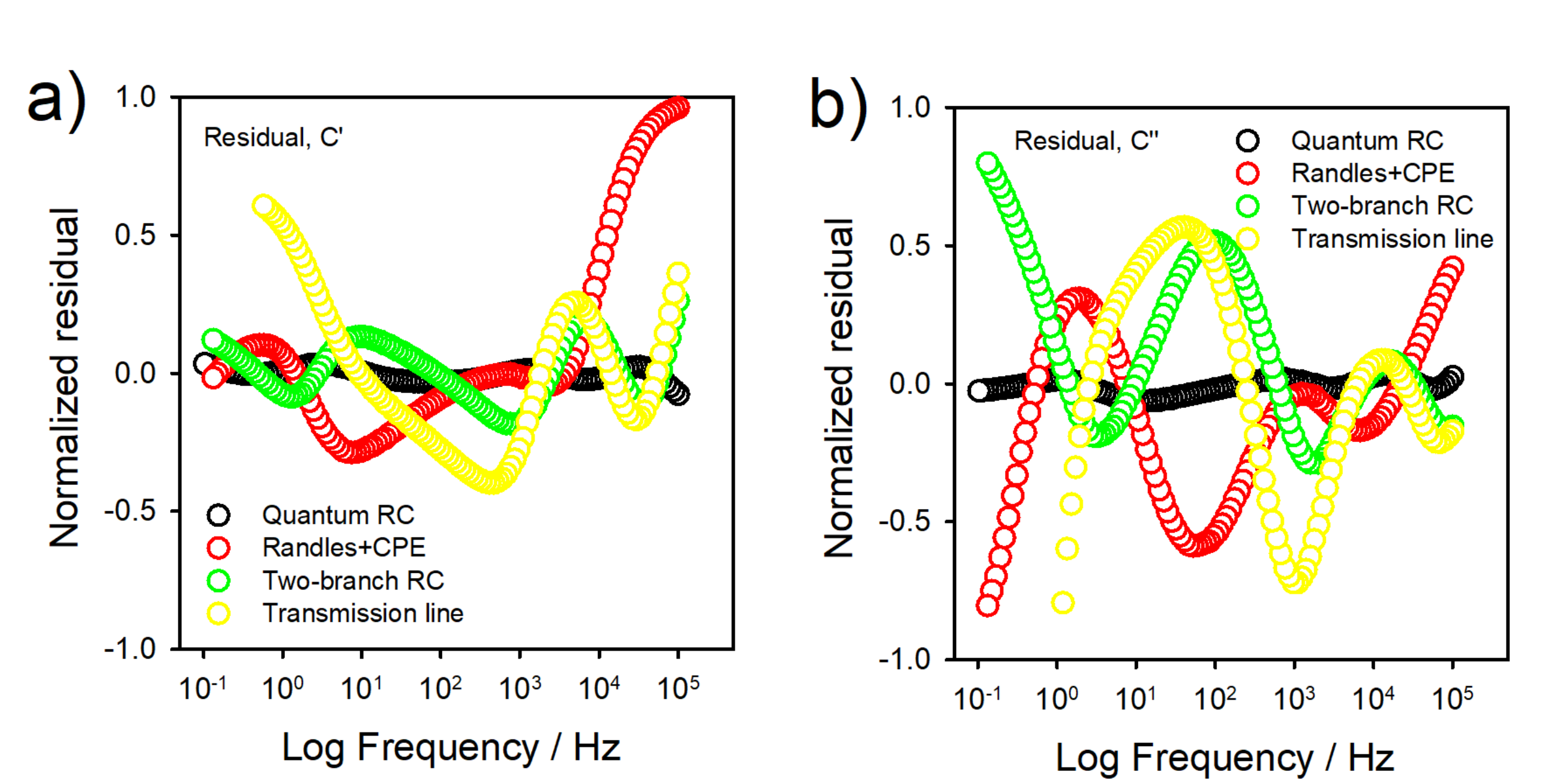

Figure S5: Normalized fitting residuals of C' and C'' for the model of the main text and the three alternative models.

S7 Attribution of the Low-Frequency Relaxation: Differential Controls

The attribution of the low-frequency relaxation observed for ox-SLG to oxidation-induced electronic states, rather than to the electrolyte, substrate, contacts, or surface heterogeneity, follows from the differential design of the experiment, in which every extrinsic element of the interface is held fixed between the pristine and oxidized measurements:

1. **Electrolyte, substrate, and contact control.** The pristine-SLG measurement constitutes the direct control for all extrinsic contributions: it was performed in the same electrochemical cell, with the same [BMIM]BF_4 batch, on the same Si/SiO_2 substrate architecture, and with the same Ti/Au contact geometry as the ox-SLG measurement, and it exhibits a single capacitive relaxation over the entire frequency window (Figure 4a of the main text). Any relaxation originating from the electrolyte, the dielectric support, or the metallic contacts would necessarily be present in both measurements. Since electrochemical oxidation is the only variable altered between the two experiments, the additional low-frequency relaxation of ox-SLG can only originate from the oxidation-induced modification of the graphene electronic structure.

2. **Charge-correlation control.** The oxidation protocol was charge-monitored (see Section S10); the emergence of the low-frequency relaxation accompanies the passage of anodic charge, tying the new process directly to the extent of oxidation rather than to any time- or handling-related artefact.

3. **Surface heterogeneity.** The Raman statistics of Section S4 show a narrow I_D/I_G distribution (IQR $\approx$ 0.03–0.04) across twelve independent locations, indicating macroscopically uniform oxidation. Heterogeneity at this level broadens, but does not create, capacitive relaxations, and cannot account for the three-orders-of-magnitude separation between the equilibrium frequencies ω_1 and ω_2 of the two processes.

4. **Timescale consistency.** The high-frequency relaxation of ox-SLG retains the equilibrium frequency and the quantized resistance of pristine SLG (Table 2 of the main text), i.e., the pristine-like channel is preserved and unaltered within the oxidized interface itself, providing an internal reference against which the new low-frequency channel is resolved.

S8 Capacitance-to-DOS Conversion, Error Propagation, and Energy Resolution of QRS

Conversion chain and units. The raw impedance $Z^*(\omega)$ [Ω] is converted to the complex capacitance $C^*(\omega) = 1/j\omega Z^*(\omega)$ [F, or F cm^{-2} after normalization by the geometric electrode area]. The equilibrium quantum capacitance $C_q(V)$ [F cm^{-2}] is read at the frequency where the capacitive semicircle closes. The areal DOS follows from $C_q = e^2\,(dN/dE)$ as $\mathrm{DOS}(V) = C_q/e^2$ [J^{-1} cm^{-2}] when the energy is expressed in joules; in the practical units used throughout this work, in which the energy axis is expressed in electronvolts and coincides numerically with the potential axis, the same quantity reads $\mathrm{DOS}(V) = C_q/e$ [eV^{-1} cm^{-2}], with C_q in F cm^{-2} and e in coulombs. The number of probed states is $N = (1/e)\int_{V_1}^{V_2} C_q\,dV$ (dimensionless), with fixed limits $V_1 = -1.5$ V and $V_2 = 0.5$ V for all data sets. For comparison with the DFT results, the measured areal DOS is normalized by the total number of probed states, DOS/N [eV^{-1}], so that experiment and theory are compared as normalized (per-state) spectral shapes rather than in absolute areal units; the DFT curves of Figure S1 are correspondingly given per carbon atom. The energy function is $E_\mu = g_s g_e N e^2/C_q$, with $g_s = g_e = 2$, which carries units of joules and is quoted throughout in eV.

Sensitivity to integration limits. Varying V_1 and V_2 by ± 0.1 V changes N by 10.4% and the assigned defect-state energies by up to 99 meV; this contribution is comparable to the overall energy resolution estimated below and is accordingly included as item (iii) in the propagated uncertainties.

Error propagation. The uncertainty of each reported DOS and E_μ value combines, in quadrature: (i) the standard errors of C_q from the equivalent-circuit fits over three independent replicates (Tables 1 and 2 of the main text); (ii) the potential-axis uncertainty, conservatively bounded by the reproducibility of the open-circuit potential across the three independent replicates (±21 mV for pristine and ±65 mV for oxidized SLG), which encompasses both electrode-to-electrode variability and drift of the Pt quasi-reference over the measurement series; and (iii) the integration-limit sensitivity above.

Energy resolution budget. The instrumental resolution of QRS combines the AC perturbation amplitude (10 mV RMS, ∼28 meV peak-to-peak), thermal broadening at 298 K ($\approx 3.5\, k_B T \approx 90$ meV), the potential step of the sweep (20 mV), and the potential-axis uncertainty bounded by the OCP reproducibility (±21 mV pristine, ±65 mV oxidized), yielding a combined resolution of ≈0.10 eV (pristine) to ≈0.12 eV (oxidized). This is comparable to the peak-position precision of the XPS analysis (±0.1 eV), while the XPS spectral linewidths (FWHM 0.8–1.8 eV) are dominated by chemical/lifetime broadening and are not directly commensurable.

S9 Quantitative XPS Analysis

Table S3 reports, for each fitted C 1s component of the pristine and oxidized samples: binding energy, FWHM, and relative area with its statistical uncertainty. The component parameters correspond to the deconvolution shown in Figure 3 of the main text; the statistical uncertainties of the relative areas were obtained by linear re-projection of the fitted components onto the raw (Shirley-subtracted) spectra, which reproduces the data with $R^2 = 0.9995$ (pristine) and $R^2 = 0.9984$ (oxidized) and amplitude scale factors within 3% of unity, confirming the internal consistency of the deconvolution. Binding-energy positions carry the ±0.1 eV calibration precision of the instrument, and an additional systematic uncertainty of ±5% (relative) applies to any composition derived from the areas through Scofield-type

quantification. The central quantitative result is the increase of the epoxy (C–O–C) fraction of the C 1s envelope from 26.6 ± 0.2% to 48.9 ± 0.3% upon oxidation, at the expense of the sp^2 C–C fraction (57.7 ± 0.1% → 47.0 ± 0.3%). The carbonyl and carboxyl components remain minor throughout and in fact decrease in relative terms (9.6 ± 0.1% → 1.5 ± 0.2% and 6.1 ± 0.1% → 2.6 ± 0.2%), which indicates that anodic oxidation under the present conditions converts the surface predominantly into epoxy functionalities; the C=O and O–C=O intensity of the pristine sample originates largely from adventitious contamination and transfer residues (Spectroscopic Characterization subsection of the Results and Discussion in the main text) and is partly consumed by the treatment. The relation between these relative areas and the defect states resolved by QRS is discussed in the Energy-State and Spectroscopic Analysis section of the main text.

Table S3: Quantitative deconvolution of the C 1s spectra of pristine and oxidized SLG (components of Figure 3 of the main text). Binding energies carry the ±0.1 eV instrumental calibration precision; relative areas are given with the statistical standard error of the linear re-projection onto the raw spectra, and compositions derived from these areas carry an additional ±5% relative systematic (Scofield) uncertainty.

Sample	Component	BE / eV	FWHM / eV	Area / % of C 1s
pristine SLG	C–C (sp^2)	284.51	1.22	57.7 ± 0.1
pristine SLG	C–O–C	285.34	1.46	26.6 ± 0.2
pristine SLG	C=O	286.42	1.81	9.6 ± 0.1
pristine SLG	O–C=O	288.92	1.81	6.1 ± 0.1
ox-SLG	C–C (sp^2)	284.43	1.57	47.0 ± 0.3
ox-SLG	C–O–C	285.36	1.71	48.9 ± 0.3
ox-SLG	C=O	287.00	0.79	1.5 ± 0.2
ox-SLG	O–C=O	288.02	1.49	2.6 ± 0.2

S10 Electrochemical Oxidation Protocol and Batch Variability

The oxidation protocol consisted of six chronoamperometric pulses of +1.7 V vs. Ag|AgCl for 45 s each, separated by 120 s rest periods at open circuit, in PB pH 3. Figure S6 shows

the current transient recorded during the final pulse of each of the three independently oxidized devices. The three independently prepared devices exhibited anodic charges of 169.25, 198.94 and 249.08 μC cm^{-2} during this pulse (Table S4), corresponding to an average of 205.8±40.4 μC cm^{-2} (RSD = 19.6%). No target charge or acceptance window was defined a priori; this dispersion is reported as the batch-to-batch reproducibility of the oxidation protocol. Batch variability is further characterized by the I_D/I_G statistics of Section S4 and the replicate standard errors of Tables 1 and 2 of the main text.

Table S4: Anodic charge passed during the final oxidation pulse for each independent device.

Device	Pulses	Charge, final pulse / μC cm^{-2}
1	6	169.25
2	6	198.94
3	6	249.08

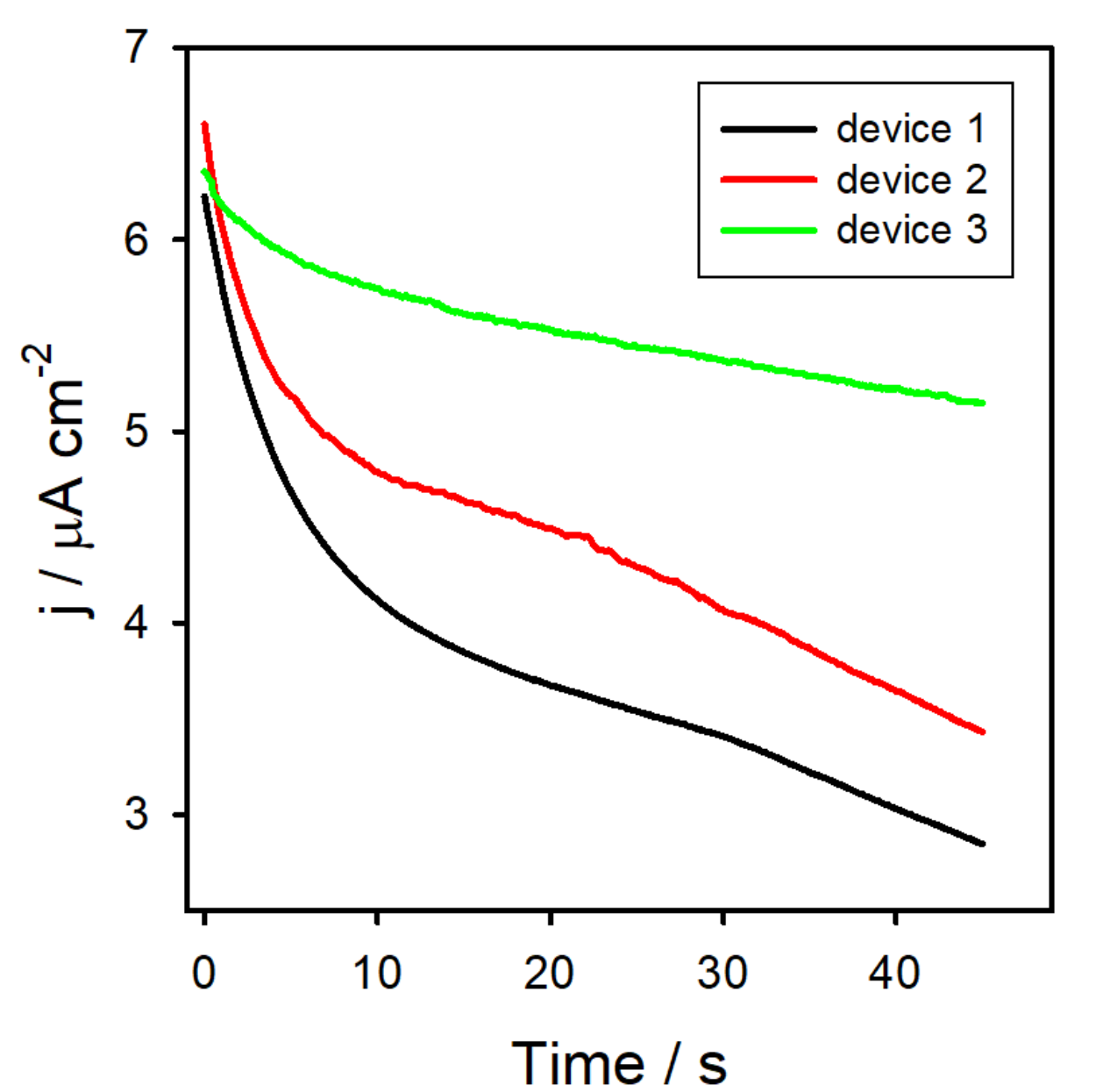

Figure S6: Representative 45-s chronoamperometric oxidation pulse (+1.7 V vs. Ag|AgCl) recorded for the three independent devices.

References

(S1) Lopes, L. C.; Pinzón, E. F.; Dias-da-Silva, G.; Feliciano, G. T.; Bueno, P. R. Electrochemical measurement of the electronic structure of graphene via quantum mechanical rate spectroscopy. *Electrochimica Acta* **2024**, *480*, 143837.

(S2) Nieto, E. F. P.; Lopes, L. C.; dos Santos, A.; Raposo, M. M. M.; Bueno, P. R. Quantum rate electrodynamics and resonant junction electronics of heterocyclic molecules. *Electrochimica Acta* **2024**, *501*, 144749.

(S3) Pinzón, E. F.; Lopes, L. C.; Fonseca, A. F. V.; Schiavon, M. A.; Bueno, P. R. Quantum rate as a spectroscopic methodology for measuring the electronic structure of quantum dots. *Journal of Materials Chemistry C* **2024**, *12*, 4606–4617.

(S4) Yan, J.-A.; Chou, M. Oxidation functional groups on graphene: Structural and electronic properties. *Physical Review B—Condensed Matter and Materials Physics* **2010**, *82*, 125403.

(S5) Lundie, M.; Šljivančanin, Ž.; Tomić, S. Electronic and optical properties of reduced graphene oxide. *Journal of Materials Chemistry C* **2015**, *3*, 7632–7641.

(S6) Savazzi, F.; Risplendi, F.; Mallia, G.; Harrison, N. M.; Cicero, G. Unravelling some of the structure–property relationships in graphene oxide at low degree of oxidation. *The Journal of Physical Chemistry Letters* **2018**, *9*, 1746–1749.

(S7) Cançado, L. G.; Jorio, A.; Ferreira, E. M.; Stavale, F.; Achete, C. A.; Capaz, R. B.; Moutinho, M. d. O.; Lombardo, A.; Kulmala, T.; Ferrari, A. C. Quantifying defects in graphene via Raman spectroscopy at different excitation energies. *Nano Letters* **2011**, *11*, 3190–3196.

(S8) Ferrari, A. C.; Robertson, J. Interpretation of Raman spectra of disordered and amorphous carbon. *Physical Review B* **2000**, *61*, 14095–14107.

(S9) Lucchese, M. M.; Stavale, F.; Ferreira, E. M.; Vilani, C.; Moutinho, M. d. O.; Capaz, R. B.; Achete, C. A.; Jorio, A. Quantifying ion-induced defects and Raman relaxation length in graphene. *Carbon* **2010**, *48*, 1592–1597.
